# All-cause and cause-specific mortality among people with bipolar disorder: a large-scale systematic review and meta-analysis

**DOI:** 10.1038/s41380-023-02109-9

**Published:** 2023-07-25

**Authors:** Taís Boeira Biazus, Gabriel Henrique Beraldi, Lucas Tokeshi, Luísa de Siqueira Rotenberg, Elena Dragioti, André F. Carvalho, Marco Solmi, Beny Lafer

**Affiliations:** 1 Bipolar Disorder Research Program, Department and Institute of Psychiatry, University of São Paulo Medical School, São Paulo, Brazil; 2 Schizophrenia Research Program (Projesq), Department and Institute of Psychiatry, University of São Paulo Medical School, São Paulo, Brazil; 3 Consultation Liaison, Department of Psychiatry, University of São Paulo Medical School, São Paulo, Brazil; 4 Research Laboratory Psychology of Patients, Families & Health Professionals, Department of Nursing, School of Health Sciences, University of Ioannina, Ioannina, Greece; 5 Pain and Rehabilitation Center, and Department of Health, Medicine and Caring Sciences, Linköping University, SE 58185 Linköping, Sweden; 6 IMPACT, The Institute for Mental and Physical Health and Clinical Translation, School of Medicine, Barwon Health, Deakin University, Geelong, Australia; 7 Department of Psychiatry, University of Ottawa, Ottawa, ON Canada; 8 Department of Mental Health, The Ottawa Hospital, Ottawa, ON Canada; 9 Ottawa Hospital Research Institute (OHRI) Clinical Epidemiology Program University of Ottawa, Ottawa, ON Canada; 10 School of Epidemiology and Public Health, Faculty of Medicine, University of Ottawa, Ottawa, ON Canada; 11 Department of Child and Adolescent Psychiatry, Charité Universitätsmedizin, Berlin, Germany

**Keywords:** Bipolar disorder, Depression

## Abstract

**Objective:**

Bipolar disorder (BD) is associated with premature mortality. All-cause and specific mortality risks in this population remain unclear, and more studies are still needed to further understand this issue and guide individual and public strategies to prevent mortality in bipolar disorder Thus, a systematic review and meta‐analysis of studies assessing mortality risk in people with BD versus the general population was conducted. The primary outcome was all‐cause mortality, whilst secondary outcomes were mortality due to suicide, natural, unnatural, and specific‐causes mortality.

**Results:**

Fifty-seven studies were included (BD; *n* = 678,353). All‐cause mortality was increased in people with BD (RR = 2.02, 95% CI: 1.89–2.16, k = 39). Specific‐cause mortality was highest for suicide (RR = 11.69, 95% CI: 9.22–14.81, k = 25). Risk of death due to unnatural causes (RR = 7.29, 95% CI: 6.41–8.28, k = 17) and natural causes (RR = 1.90, 95% CI: 1.75–2.06, k = 17) were also increased. Among specific natural causes analyzed, infectious causes had the higher RR (RR = 4,38, 95%CI: 1.5–12.69, k = 3), but the analysis was limited by the inclusion of few studies. Mortality risk due to respiratory (RR = 3.18, 95% CI: 2.55–3.96, k = 6), cardiovascular (RR = 1.76, 95% CI: 1.53–2.01, k = 27), and cerebrovascular (RR = 1.57, 95% CI: 1.34–1.84, k = 13) causes were increased as well. No difference was identified in mortality by cancer (RR = 0.99, 95% CI: 0.88–1.11, k = 16). Subgroup analyses and meta-regression did not affect the findings.

**Conclusion:**

Results presented in this meta-analysis show that risk of premature death in BD is not only due to suicide and unnatural causes, but somatic comorbidities are also implicated. Not only the prevention of suicide, but also the promotion of physical health and the prevention of physical conditions in individuals with BD may mitigate the premature mortality in this population. Notwithstanding this is to our knowledge the largest synthesis of evidence on BD-related mortality, further well-designed studies are still warranted to inform this field.

## Background

Bipolar disorder (BD) is a chronic, often progressive, and disabling disorder that has peak and median age at onset at age 19.5 and 33 years old [1], and affects roughly one percent of the global population, regardless of sex, ethnicity, or social income [2–4]. BD is one of the most incapacitating diseases among young and working age adults and is strongly associated with lower productivity levels, functional and social impairment, increased rates of clinical and psychiatric comorbidities and premature mortality [5, 6].

The association between mental disorders and mortality is complex, as the relative risk is the highest for suicide, but the most frequent causes of the death are the physical comorbidities associated to it (e.g., metabolic, cardiovascular, cerebrovascular, infectious, and respiratory diseases) [4, 5]. Although the current literature has focused on major depression [7] and schizophrenia [8], a raising body of evidence have been published suggesting the association between BD and premature death.

Initial studies suggested that higher mortality levels in BD patients were solely attributed to suicide [9, 10]. New research, however, indicates an increase in mortality by natural causes. In fact, compared to the general population, individuals with BD are at a greater risk of physical diseases which contributes to increase the mortality rates [11, 12].

At least part of the increased risk for natural causes of death in people with BD and other mental disorders is due to unhealthy lifestyles, adverse effects of psychiatric medications, limited access to the health care system, and disparities in diagnosis and treatment of comorbid clinical diseases [13]. Additionally, intrinsic mechanisms associated to BD such as inflammatory and oxidative processes, and genetic vulnerability are in turn associated with mortality [11, 14, 15].

To our knowledge, three reviews investigated excess mortality in individuals with BD. In 1998, Harris and Barraclough found an increase of mortality due to violent causes, but only six studies were included (*N* = 3801) [16]. In their analysis, mortality for suicide was 11 times more prevalent in BD compared to general population [16]. In 2009, Roshanaei-Moghaddam and Katon included 17 studies (*N* = 331,000), showing that BD were associated with increased premature mortality due to natural causes, mainly cardiovascular disease [11]. Finally, in 2015, Hayes et al. included 31 studies (*N* = 305,859) and found a twofold increase in the overall mortality in BD compared to the general population [17].

Previous reviews had some limitations as (a) screening in a single database [11], (b) including the absence of exploring all sources of heterogeneity, (c) inclusion of a small range of size effect (SMR), (d) the authors did not perform a quality assessment [17–19]. Due to the above-cited topics and since more studies have been published since the last evidence synthesis, an updated meta-analysis assessing the relative risk of all-cause and cause-specific mortality in those with BD versus the general population is needed. In this review, we aimed to fill this gap and conducting a comprehensive systematic review and meta-analysis on all-cause and cause-specific mortality relative risk in people with BD compared with any control group.

## Methods

### Protocol and registration

The reporting of this systematic review and meta-analysis was guided by the standards of Preferred Reporting Items for Systematic Reviews and Meta-analyses (PRISMA) [20] and Meta-analyses Of Observational Studies in Epidemiology (MOOSE) [21]. The PROSPERO protocol was registered on July 14, 2020 (CRD42020192217).

### Eligibility criteria

Inclusion criteria were: (1) observational studies that compared risk of any cause of mortality between (2) patients with BD (defined according to DSM/ICD criteria or clinical charts) and a control group, (3) aged 15 years or older or providing estimates in such an age group. No language restrictions were applied. The exclusion criteria were the following: (1) studies that did not specify diagnostic criteria or whose individuals with BD could not be separated from a broader sample (i.e., a group of subjects with mood disorders), (2) studies with less than 50 patients, (3) studies that included solely individuals younger than 15 years old, (4) studies that included only individuals in a very specific subgroup, because this subgroup would not be a representative sample of bipolar disorder (i.e., prison population), (5) duplicated samples (i.e., a smaller sample from a larger cohort published elsewhere), (6) randomized controlled trials, (7) systematic or non-systematic reviews and meta-analysis.

### Information sources

Studies were selected by searching in the following databases: PubMed/Medline, Embase, Web of Science and PsycInfo, up to July 20th, 2021. References and bibliography list of relevant papers were examined to track down potential studies that were not identified in the initial search.

### Search strategy

Studies were screened using the terms related: (1) to bipolar disorders (e.g., bipolar disorder, bipolar affective disorder, bipolar illness, bipolar depression, and mood disorder), (2) to mortality (e.g., mortality, fatal outcome, life expectancy and death) and (3) to estimates (e.g., standardized mortality ratio or SMR, hazard ratio or HR, odds ratio or OR, and mortality rate ratio or MRR). A complete description of search terms used in each database can be found in Supplementary Table S1. After the database search, duplicated papers and samples were removed, and the study selection started.

### Study selection

The studies were first screened based on title and abstract by authors TB and LSR. Selected studies were analyzed in full text by two pairs independently (TB and LSR, LT and GHB) and those that did not met inclusion criteria were removed. Discrepancies in any phase were discussed between the authors TB, GHB, MS, ED and BL until a consensus was reached. The remaining papers were then included in the meta-analysis. The software Covidence was used to conduct the processes from studies screening to data extraction [22].

### Data extraction

Data extraction was conducted in pairs independently by TB, LSR, LT and GHB and discrepancies were discussed between authors until a consensus was reached.

We extracted the author, year of publication, country, sex, study design, representativeness, setting (inpatient, outpatient, mixed), BD diagnostic criteria, control group, sample size, outcome, estimates of mortality risk with dispersion measure, adjusted versus non-adjusted analyses, and the information needed to conduct quality assessment.

### Statistical analyses

We used RR with 95% CI to describe the summary results of meta-analysis. A RR superior to 1 indicating a higher mortality in individuals with BD, whereas an RR inferior to 1 indicating an increased mortality in the general population or control group [23]. We pooled together SMR, MRR, RR, OR, and HR given that as the event rate was rare, i.e., less frequent than 10%, and the study design, population, comparison, and outcome were comparable [23]. Similar statistical method was used in other meta-analyses regarding mortality in mental diseases groups [8, 24].

Whenever feasible, subgroup analyses were conducted to assess the mortality categorical outcomes stratified by representativeness (whether the sample was representative of the whole population), sample type (outpatient, inpatient or community), study design (prospective, retrospective or case-control), location (Africa, Asia, North America, Europe or other), BD diagnostic criteria (DSM, ICD or other) the use of a structured interview to assess patients adjustment by substance use disorder (SUD) and time-at-risk.

Regarding representativeness, a sample was considered representative when used a populational-based design. It was not considered representative when included inpatients or only individuals with any specific condition (i.e., only BD patients using lithium, or individuals with pre-existing somatic disorders).

We used a random-effects model to calculate the summary RR, based on DerSimonian and Leird method as we could not assume that the same true effect was present in all studies [25]. Statistical analysis was conducted using Comprehensive Meta-Analysis (CMA) version 3. Finally, we calculated the prediction intervals to each meta-analysis to assess the distribution of true effects [26].

### Heterogeneity and publication bias

Heterogeneity was assessed using the Q-test and the I^2^ statistics. Publication bias was calculated using Egger’s regression test and visually assessed by funnel plot [27, 28].

### Risk of bias of individual studies

The risk of bias of the included articles was assessed by two independent reviewers independently (TB and GHB) according to the Newcastle-Ottawa Scale (NOS). This instrument is used to evaluate the quality of non-randomized studies in meta-analyses. The NOS consists of 8 items divided into 3 categories: selection, comparability, and outcome (cohort studies) or exposure (case-control studies). Studies scoring 6 or more are considered high-quality, while those scoring 5 or less are considered of low quality [29].

### Meta-regression

Meta-regression was performed to assess the influence of confounding factors affecting results and heterogeneity. The continuous variables chosen to be assessed trough meta- regression were sample size, percentage of females, mid-point year of the study, mean follow- up years and NOS score. Since mortality may be affected by social conditions, we additionally included social developing indicators of the countries where the studies were conducted. Hence, both Social Development Index (SDI) and Human Development Index (HDI) were also used as moderators in the meta-regression [30, 31].

### Level of certainty of the results

GRADE is a practical to tool evaluate the certainty in the evidence for systematic review authors and decision-makers. Herein, GRADE was used to classify the confidence level in the RR estimates as high, moderate, low, or very low [32].

## Results

### Study selection

**Fig. 1:**
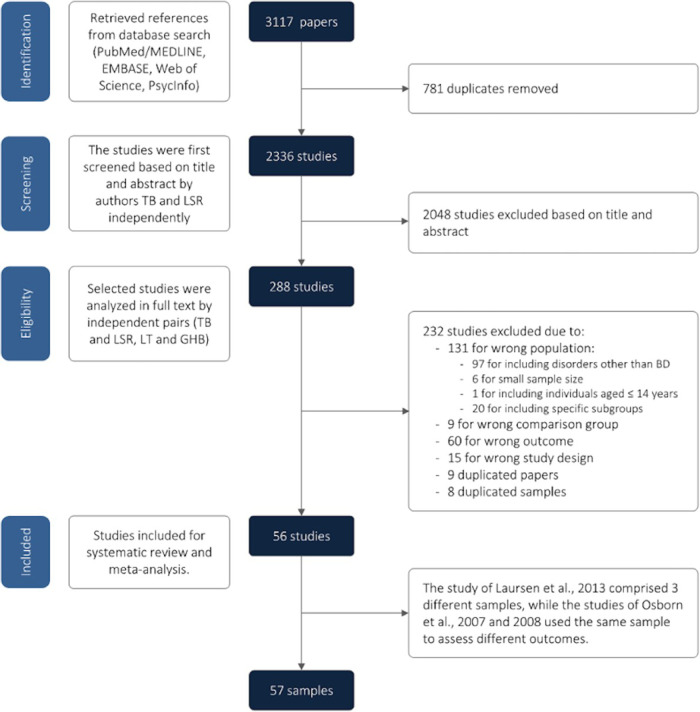
PRISMA flowchart. PRISMA Flowchart for elegible and included studies.

Database search yielded 2336 manuscripts eligible for screening, after the removal of duplicates. Next, 2048 articles were excluded based on title and abstract, and 288 papers were retrieved. After the full-text review, 232 studies were subsequently excluded, resulting in 56 studies included in this meta-analysis. However, the study of Laursen et al., 2013 [33] was comprised of three different samples, while the studies of Osborn et al. 2007 [34] and 2008 [35] used the same sample to assess different outcomes. Hence, the final analysis was composed by 57 samples [36–90]. Figure 1 presents the PRISMA flow diagram for study selection and PRISMA Checklist can be found in suplementar material (Table S6)

The main reasons for exclusion were due to population characteristics (mainly when the population was a specific subgroup), or when the statistical analyses for BD as a separate diagnosis. Overall, 124 papers reported data from a broad group, as severe mental illness, mood disorders or affective disorders, rather than specifically on subjects with BD. Other seven studies included a very specific group of bipolar patients, for instance veterans, long-stay psychiatric patients and people without a fixed address. Other nine studies included only individuals with BD but compared physically healthy individuals with BD with subjects with BD and a comorbid organic disease [91–99]. Eight studies reported data from duplicated samples and were not included in the meta-analysis [100–107]. In these cases, we used the most recent and/or the larger sample to choose the included study. Finally, 60 studies did not assess mortality. All excluded articles and the reason for exclusion can be found in the Supplementary Table S2.

### Studies characteristics

The 57 samples included in the analysis comprised 678 353 individuals with BD. Assessment data ranged from 1934 to 2016. Ten studies did not report data separately from men and women [108–117] and three studies only included males [118–120]. Sex analysis included 46 studies with males (k = 47 samples, 262 971 individuals) and 43 studies with females (k = 44 samples, 342,493 individuals). As most of the included studies were observational with a longitudinal follow-up with a wide range (1 to 52 years) and there were not at least ten studies reporting mean age with the same outcome, it was not possible to calculate the mean age to use as a moderator in the statistical analysis. The sociodemographic characteristics of the included studies are compiled in Table 1. Additional characteristics are available in Supplementary Table S3.

**Table 1:** Demographic characteristics of the included studies.

Study	Location	Sample size (men/woman)	Recruitment Date^a^	Outcome	Diagnostic criteria	Sample type	Represen- tative^b^	Structured interview^c^	NOS^d^	Type of mortality assessed
Ahrens 1995 [36]	Multicentric	440 (251/189)	1967–1992	SMR	NA	Outpatients	No	Yes	5	All-cause, suicide, cardiovascular
Ajetunmobi 2013 [37]	United Kingdom	5778 (2159/3619)	1986–2010	SMR	ICD-10	Inpatients	No	No	7	All-cause, natural, unnatural, suicide, cardiovascular, cancer, cerebrovascular
Almeida 2016 [38]	Australia	288 (288/0)	1996–2011	HR	ICD-8 and 9	Community	Yes	No	8	Suicide
Angst 2002 [39]	Switzerland	220 (73/147)	1959–1997	SMR	ICD-8	Inpatients	No	Yes	7	All-cause, suicide, cardiovascular, cancer
Angst 2005 [40]	Switzerland	220 (73/147)	1959–2003	SMR	ICD-9	Inpatients	No	Yes	6	All-cause, suicide, cardiovascular, cancer, cerebrovascular
Angst 2013 [41]	Switzerland	190 (132/68)	1959–2009	SMR	ICD-10	Inpatients	No	No	7	All-cause, suicide, cardiovascular, cancer, cerebrovascular
Black 1987 [42]	United States	586 (NA/NA)	1970–1983	SMR	DSM III	Inpatients	No	No	6	All-cause, natural, unnatural, cerebrovascular
Bratfos 1968 [9]	Norway	207 (93/114)	1952–1963	SMR	Langfeldt	Inpatients	No	Yes	6	All-cause
Callaghan 2014 [43]	United States	76098 (30978/45120)	1990–2005	SMR	ICD-9	Inpatients	No	No	7	Cardiovascular, respiratory, cancer
Castagnini 2013 [44]	Denmark	3200 (NA/NA)	1995–2008	SMR	ICD-10	Inpatients	No	No	6	All-cause, natural, unnatural, suicide, cardiovascular, respiratory, cancer, infectious, cerebrovascular
Chang 2010 [45]	United Kingdom	2699 (1126/1573)	2007–2010	SMR	ICD-10	Inpatients	No	No	7	All-cause, unnatural, suicide
Chang 2012 [46]	United Kingdom	1542 (574/968)	2007–2010	HR	ICD-10	Community	Yes	No	7	All-cause, unnatural, suicide
Chen 2010 [15, 47]	Taiwan	1581 (780/801)	1996–2004	HR	ICD-9	Inpatients	No	No	7	All-cause, natural, unnatural
Chen 2020 [87]	Taiwan	46490 (23321/23169)	2001–2016	SMR	ICD-9 and 10	Inpatients	No	No	8	Sudden cardiac death
Choi 2019 [48]	South Korea	481 (214/267)	2005–2012	HR	ICD-10	Inpatients	No	No	8	Suicide
Crump 2013 [49]	Sweden	6618 (2700/3918)	2003–2009	HR	ICD-10	Community	Yes	No	_8_	All-cause, natural, unnatural, suicide, cardiovascular, cancer, cerebrovascular
Dutta 2007 [50]	United Kingdom	235 (102/133)	1965–1999	SMR	DSM IV	Inpatients	No	Yes	6	All-cause, suicide, cardiovascular, respiratory, cancer, infectious
Fekadu 2015 [51]	Ethiopia	346 (193/153)	1998–2012	SMR	SCAN	Community	Yes	No	7	All-cause
Fiedorowicz 2009 [52]	United States	435 (185/250)	1978–2003	HR	RDC	Outpatients	Yes	Yes	7	Cardiovascular
Gale 2012 [53]	Sweden	31 (31/0)	1950–2004	HR	ICD-8, 9 and 10	Inpatients	No	Yes	8	Cardiovascular
Guan 2013 [54]	Netherlands	2077 (927/1150)	1999–2009	HR	DSM-IV	Community	Yes	No	7	All-cause, suicide, cancer
Hayes 2017 [55]	United Kingdom	17314 (7139/10202)	2000–2014	HR	ICD-10	Community	Yes	No	7	All-cause, suicide, cardiovascular
Hjorthøj 2015 [56]	Denmark	6799 (2455/4344)	1969–2011	SMR	NA	Community	Yes	No	8	All-cause
Hoang 2011 [57]	United Kingdom	75720 (29534/46186)	1999–2006	SMR	ICD-9 and 10	Inpatients	No	No	_6_	All-cause, natural, unnatural, cardiovascular, respiratory, cerebrovascular
Hoang 2013 [58]	United Kingdom	14017 (5747/8270)	2006–2008	SMR	ICD-10	Inpatients	No	No	6	All-cause
Høye 2016 [59]	Norway	845 (331/514)	1980–2012	SMR	ICD-9 and 10	Inpatients	No	No	7	All-cause, suicide, cardiovascular, cancer
Kay 1977 [60]	Sweden	192 (84/108)	1958–1970	SMR	NA	Outpatients	Yes	Yes	5	All-cause
Kim 2018 [61]	South Korea	1874 (588/1286)	2002–2013	HR	ICD-10	Community	Yes	No	8	All-cause, suicide
Kodesh 2012 [62]	Israel	5732 (2539/3193)	2003–2009	MRR	ICD-9	Community	Yes	No	8	All-cause
Laursen 2007 [90]	Sweden	11648 (NA/NA)	1973–2001	MRR	ICD-8 and 10	Inpatients	No	No	8	Suicide, cardiovascular, respiratory, cancer
Laursen 2009 [63]	Denmark	NA (NA/NA)	1994–2007	MRR	ICD-8 and 10	Inpatients	No	No	8	Cardiovascular
Laursen 2011 [88]	Denmark	6215 (NA/NA)	1995–2007	MRR	ICD-8 and 10	Community	Yes	No	8	Natural
Study	Location	Sample size (men/woman)	Recruitment Date^a^	Outcome	Diagnostic criteria	Sample type	Represen- tative^b^	Structured interview^c^	NOS^d^	Type of mortality assessed
Laursen 2013a [64]	Denmark	11101 (4280/6821)	2000–2007	SMR	ICD-9 and 10	community	Yes	No	8	All-cause, natural, unnatural, cardiovascular, cerebrovascular
Laursen 2013b [64]	Finland	9919 (4489/5430)	2000–2007	SMR	ICD-9 and 10	Community	Yes	No	8	All-cause, natural, unnatural, cardiovascular, cerebrovascular
Laursen 2013c [64]	Sweden	18355 (7367/10988)	2000–2007	SMR	ICD-9 and 10	Community	Yes	No	8	All-cause, natural, unnatural, cardiovascular, cerebrovascular
Lomholt 2019 [65]	Denmark	23092 (9510/13582)	1995–2014	SMR	ICD-8 and 10	Community	Yes	No	8	All-cause
Medici 2015 [66]	Denmark	15334 (NA/NA)	1995–2012	SMR	ICD-10	Community	Yes	No	8	All-cause
Mohamed 2019 [67]	United States	41362 (21632/19730)	2004–2014	OR	ICD-9	Inpatients	No	No	8	Cardiovascular
Newman 1991 [68]	Canada	1429 (543/886)	1976-1985	SMR	ICD-9	Community	Yes	No	8	All-cause, suicide
Norton 1984 [69]	United Kingdom	791 (NA/NA)	1967–1977	SMR	Feighner	Community	No	No	7	All-cause, suicide, cardiovascular, cancer
Osborn 2007/2008 [70, 89]	United Kingdom	10742 (5725/5017)	1987–2002	HR	Oxmis	Community	Yes	No	8	Cardiovascular, cerebrovascular
Ösby 2001 [71]	Sweden	15386 (6578/8808)	1973–1995	SMR	ICD-8 and 9	Inpatients	No	No	8	All-cause, natural, unnatural, suicide, cardiovascular, respiratory, cancer, infectious, cerebrovascular
Pan 2017 [87]	Taiwan	77859 (33415/44444)	2003–2007	SMR	ICD-9	Community	Yes	No	7	All-cause, natural, unnatural, cerebrovascular
Pan 2020 [73]	Taiwan	103709 (43843/59866)	2005–2013	SMR	ICD-9	Community	Yes	No	6	All-cause, natural, unnatural, cerebrovascular
Park 2019 [74]	South Korea	3470 (1706/1764)	2002–2013	SMR	ICD-10	Community	Yes	No	7	Suicide
Ramsey 2013	United States	2519 (1268/1251)	1980–2007	OR	DSM III	Community	Yes	No	7	All-cause
Saku 1995 [76]	Japan	187 (119/68)	1948–1985	SMR	DSM III	Inpatients	No	No	6	All-cause, cancer
Schaffer 2014 [77]	Canada	170 (96/74)	1998–2010	OR	Coroner	Community	Yes	No	7	Suicide
Schneider 2001 [78]	Germany	74 (24/50)	1983–1993	SMR	DSM III, ICD-9	Inpatients	No	Yes	7	All-cause, natural, unnatural, cerebrovascular
Schulman-Marcus 2016 [79]	United States	16913 (9353/7560)	2001–2012	OR	ICD-9	Inpatients	No	No	8	Cardiovascular
Sharma 1994 [80]	United Kingdom	472 (NA/NA)	1970–1987	SMR	DSM III	Inpatients	No	No	4	Suicide, cardiovascular, respiratory
Tsuang 1980 [81]	United States	92 (34/58)	1934–1974	SMR	Iowa 500 cohort	Inpatients	No	No	7	All-cause, natural, unnatural, suicide, cardiovascular, cancer, infectious, cerebrovascular
Vinogradova 2010 [82]	United Kingdom	159 (63/96)	2000–2005	HR	Other^e^	Community	No	No	6	All-cause
Webb 2014 [83]	Sweden	15337 (NA/NA)	1973–2009	RR	ICD-8, 9 and 10	Community	Yes	No	8	Suicide
Weeke 1987 [84]	Denmark	2662 (2662/0)	1950–1957	SMR	ICD-8	Inpatients	No	No	5	All-cause, suicide, cardiovascular
Westman 2013 [85]	Sweden	17101 (NA/NA)	1987–2006	SMR	ICD-9 and 10	Inpatients	No	No	7	All-cause, natural, unnatural, suicide, Cardiovascular, cancer, cerebrovascular
Yeh 2019 [86]	United States	213 (102/111)	2000–2013	OR	ICD-9	Community	Yes	No	8	Suicide

*HR* hazard ratio, *MRR* mortality rate ratio, *NA* not available, *OR* odds ratio, *RR* risk ratio, *SCAN* Clinical Assessment in Neuropsychiatry, *SMR* standardized mortality ratio.

^a^Data since the first individual from the last individual entered and do not necessarily correspond to the data of the last assessment of mortality.

^b^Whether the sample is representative from the general population or not.

^c^Whether the researchers used a structured interview to recruit individuals to the study.

^d^The NOS (Newcastle-Ottawa Scale) is used to assess the risk of bias.

^e^Standard computer codes (Read codes) for general practice in the UK.

The samples were obtained from 16 different countries, mainly from western Europe (k = 35), followed by North America (k = 10) and Asia (k = 9). Africa [121] and Oceania [118] were represented by one study each, while another study was multicentric [122]. Most of studies used retrospective (k = 28) or prospective cohorts (k = 26), while few of them were case-control studies (k = 3). Studies included samples of inpatients (k = 28), community (k = 26) or outpatient (k = 3). Follow-up time ranged from 1 to 52 years.

Regarding estimates, 35 studies reported SMR, while 12 used HR, five used OR, four used MRR and one used RR. From the 58 samples included in the final analysis, 26 were considered representative of the population, while 31 were considered not representative.

### Risk of bias of individual studies

The scores in the NOS ranged from four to eight in the included studies. Only four up to 56 studies were considered at high risk of bias [108, 120, 122, 123]. The remaining studies scored six or more and were considered at a low risk of bias. Individual risks of each study are presented in Table 1, whereas a detailed scoring is described in the Supplementary Table S4. Supplementary Figure S1 shows a visual distribution of risk of bias of individual studies and Supplementary Figure S2 summarizes the distribution of biases regarding all included studies.

### Overall mortality

All-causes mortality was assessed in 39 samples comprising 450 397 individuals with BD. The pooled RR for overall mortality in the total population was RR = 2.02 (95%CI 1.89–2.16, *p* < 0.001, k = 39). The RR was similar between men (RR = 2.27, 95%CI 2.13–2.43, *p* < 0.001, k = 23) and women (RR = 2.26, 95%CI 2.08–2.46, *p* < 0.001, k = 22).

**Fig. 2:**
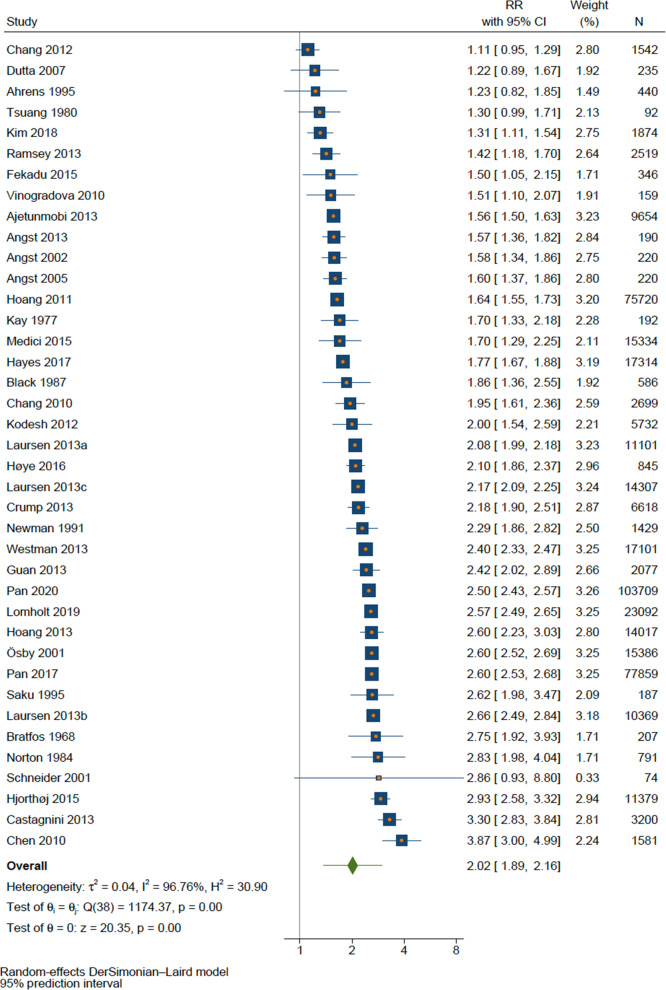
Forest plot of all-cause mortality.

There was a high between-study heterogeneity, either for total population (Q = 1174.2, *p* < 0.001, I^2^ = 96.8%) or sex analysis of men (Q = 180.8, *p* < 0.001, I^2^ = 87.8%) and women (Q = 317.5, *p* < 0.001, I^2^ = 93.4%). The forest plot for all-cause mortality is shown in Fig. 2.

Although a high heterogeneity was found, when prediction interval was calculated, the true effect size remained significant and ranged from 1.37 in some populations to 2.98 in others, regardless the sex (1.73 to 2.98 for males and 1.57 to 3.27 for females). The results and graphs of prediction interval are presented in Supplementary Fig. S3.

A significant publication bias was found in the analysis of overall mortality (Egger’s test *p* = 0.036). Funnel plot showed that most of the studies presents a low standard error, which is a consequence of the large sample sizes of the included studies. However, there is a homogeneous distribution between results that does not favor any group, whether the general population or BD (Supplementary Fig. S4). Both Egger’s test and forest plots revealed no publication bias for male (*p* = 0.360) or female (*p* = 0.328). Summary results for all-cause mortality are presented in Table 2.

**Table 2:** Summary of results.

Sex	K	N	Effect size [95%CI]	I^2^ (%)	Egger’s test *p* value	GRADE
All-cause mortality						
Total	39	450397	2.02 [1.89–2.16]	96.8	0.036	⨁⨁⨁◯
Male	23	158752^a^	2.27 [2.13–2.43]	87.8	0.360	⨁⨁⨁◯
Female	22	215505^a^	2.26 [2.08–2.46]	93.4	0.328	⨁⨁⨁◯
Mortality by natural causes						
Total	17	354417	1.91 [1.76–2.07]	96.1	0.588	⨁⨁◯◯
Male	11	103783^a,d^	2.03 [1.88–2.19]	87.9	0.893	⨁⨁⨁◯
Female	12	138050^a,d^	2.05 [1.95–2.16]	71.5	0.848	⨁⨁⨁◯
Mortality by unnatural causes						
Total	17	349744	7.29 [6.42–8.28]	94.1	0.128	⨁⨁⨁⨁
Male	10	103783^a^	6.69 [5.85–7.66]	87.3	0.454	⨁⨁⨁⨁
Female	11	138050^a^	9.33 [8.07–0.78]	86.1	0.082	⨁⨁⨁⨁
Mortality by suicide						
Total	25	180210	11.6 [9.23–14.81]	94.6	0.103	⨁⨁⨁⨁
Male	11	47568^b,e,h^	14.02 [11.16–17.60]	87.1	0.146	⨁⨁⨁⨁
Female	11	71175^b,e^	17.53 [10.76–28.54]	97.5	0.258	⨁⨁⨁⨁
Mortality by cardiovascular causes						
Total	27	387963^c^	1.76 [1.53–2.02]	98.0	0.528	⨁⨁◯◯
Male	13	96411^b^	1.82 [1.69–1.97]	73.3	0.735	⨁⨁◯◯
Female	14	154499^c^	1.69 [1.44–1.99]	95.4	0.637	⨁⨁◯◯
Mortality by cerebrovascular causes						
Total	13	165787	1.57 [1.34–.84]	85.6	0.931	⨁⨁◯◯
Male	7	56494	1.70 [1.54–1.88]	0.0	0.434	⨁⨁◯◯
Female	7	77620	1.70 [1.30–2.23]	90.9	0.254	⨁⨁◯◯
Mortality by infectious causes						
Total	3	91341^f^	4.38 [1.51–12.70]	94.7	NA**	⨁⨁◯◯
Male	2	6680	3.24 [1.90–5.52]	0.0	NA**	⨁⨁◯◯
Female	2	8941	2.62 [1.61–4.28]	0.0	NA**	⨁⨁◯◯
Mortality by respiratory causes						
Total	6	106661^g^	3.18 [2.56–3.97]	77.4	0.200	⨁⨁⨁◯
Male	5	67192^b^	3.29 [2.37–4.58]	94.7	0.606	⨁⨁⨁◯
Female	5	100247^b^	2.86 [2.05–3.98]	95.6	0.507	⨁⨁⨁◯
Mortality by cancer						
Total	16	203181	0.99 [0.88–1.11]	75.0	0.854	⨁⨁◯◯
Male	9	73004^b^	0.99 [0.93–1.06]	0.0	0.681	⨁⨁◯◯
Female	9	104805^c^	1.03 [0.87–1.21]	79.2	0.901	⨁⨁◯◯

Legend. K, number of samples; N, sample size; The following authors did not report sample size: ^a^Black 1987, Laursen ^b^2007, ^c^2009 and (d) 2011, and ^e^Castagnini 2013; Results of the following studies were excluded from analysis due to excessive asymmetry in the 95%CI: ^f^Tsuang 1980, ^g^Callaghan 2014 and ^h^Ahrens 1995; * Based on Cochrane’s GRADE (Grading of Recommendations Assessment, Development and Evaluation). ** Not enough studies to calculate. GRADE: ⨁◯◯◯: very low; ⨁⨁◯◯: Low; ⨁⨁⨁◯: Moderate; ⨁⨁⨁⨁: High.

### Mortality for specific causes

**Fig. 3:**
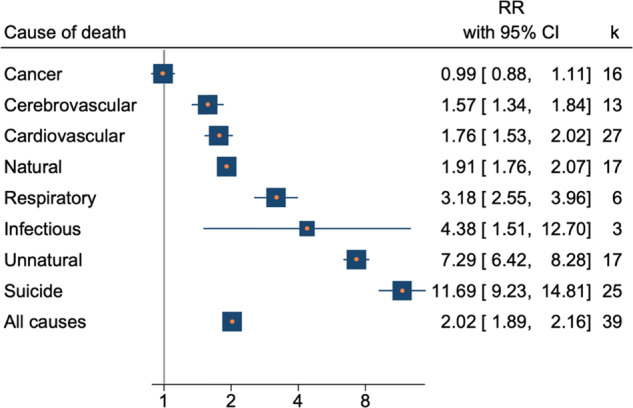
Forest plot of summary results.

Mortality also was increased for infectious (RR = 4.38, 95%CI 1.51–12.70, *p* = 0.007, k = 3), respiratory (RR = 3.18, 95%CI 2.55–3.96, *p* < 0.001, k = 6), cardiovascular (RR = 1.76, 95%CI 1.53–2.02, *p* < 0.001, k = 27), and cerebrovascular (RR = 1.57, 95%CI 1.34–1.84, p < 0.001, k = 13), and causes. Mortality by cancer was the only specific cause that was not elevated in BD when compared to general population (RR = 0.99, 95%CI 0.88–1.11, *p* = 0.894, k = 16), as shown in Fig. 3. When stratified by sex, results were not significant for cancer but remained significant for all other causes of mortality. Although mortality by infectious (k = 3) and respiratory (k = 6) causes were found to be nearly twofold higher than cardiovascular (k = 27) and cerebrovascular (k = 13), their results came from a smaller number of studies, making a comparison not reliable.

A higher SMR was observed for suicide and unnatural causes in BD when compared to general population. RR for suicide was 11.69 (95%CI 9,22–14,81, *p* < 0.001, *k* = 25), When stratified by gender, suicide SMR was higher in woman (17.52 95%CI, 10.76–28.54, *p* < 0.001, *k* = 11) than in men (14,02, 95%CI 11.16–17.60, *p* < 0.001, *k* = 11). The SMR total for unnatural causes was 7,29 (IC95% 6.42-8.28, *p* < 0.001, *k* = 17). For males the RR for unnatural causes was 6.69 (95%CI 5.85–7.66, *p* < 0.001, *k* = 10), and for females the RR was 9.33 (8.07–10.78, *p* < 0.001, *K* = 11).

Only four of the main results were not considered statistically heterogeneous: mortality from cancer (Q = 7.9, *p* = 0.443, I^2^ = 0.0%), cerebrovascular (Q = 4.9, *p* = 0.559, I^2^ = 0.0%) and infectious (Q = 0.4, *p* = 0.521, I^2^ = 0.0%) causes in men, and mortality from infectious causes in women (Q = 0.3, *p* = 0.589, I^2^ = 0.0%). None of the meta-analyses of mortality for specific causes were found to present publication bias.

Prediction intervals showed that the range of the true effects remained significant only for mortality from natural causes, unnatural causes, and suicide. Cardiovascular and cerebrovascular causes were homogeneous distributed only in men with BD. Summary results for specific mortality are presented in Table 2.

### Meta-regression and subgroup analysis

There was no difference in mortality between subgroups when analyzed by location, sample type, representativeness, study design, diagnostic criteria, use of structured interview, adjustment by substance use disorder and adjustment by time at risk (Table 3).

**Table 3:** Results from subgroup analysis.

Group	Sub-group	Population	Number of studies (k)	Risk ratio [95% CI]	*I*^2^ (%)	Group	Sub-group	Population	Number of studies (k)	Risk ratio [95% CI]	*I*^2^ (%)
Location	Africa	Total	1	NA	NA	Study design	Case–control	Total	1	NA	NA
		Male	0	NA	NA			Male	1	NA	NA
		Female	0	NA	NA			Female	1	NA	NA
	Asia	Total	6	2.33 [2.07–2.62]	93.8		Prospective	Total	21	2.02 [1.84–2.21]	94.0
		Male	5	2.49 [2.36–2.63]	46.3			Male	9	2.27 [2.02–2.56]	84.7
		Female	5	2.67 [2.44–2.93]	73.1			Female	10	2.32 [2.11–2.55]	78.4
	Europe	Total	26	2.02 [1.85–2.20]	97.2		Retrospective	Total	17	1.95 [1.76–2.17]	98.1
		Male	15	2.25 [2.06–2.46]	89.5			Male	13	2.21 [2.04–2.41]	89.4
		Female	13	2.17 [1.94–2.42]	94.4			Female	11	2.13 [1.87–2.43]	96.0
	North America	Total	5	1.83 [1.40–2.39]	81.9	Diagnostic criteria	DSM	Total	5	1.83 [1.37–2.46]	86.4
		Male	2	1.99 [1.53–2.59]	12.5			Male	3	1.93 [1.45–2.58]	30.6
		Female	3	2.14 [1.73–2.64]	0.0			Female	3	1.95 [1.09–3.49]	78.9
	Other	Total	1	NA	NA		ICD	Total	25	2.07 [1.91–2.24]	97.8
		Male	1	NA	NA			Male	17	2.30 [2.15–2.47]	90.0
		Female	1	NA	NA			Female	15	2.31 [2.11–2.54]	95.2
Sample type	Community	Total	20	2.05 [1.90–2.21]	95.8		Other	Total	6	1.90 [1.40–2.58]	74.8
		Male	11	2.25 [2.08–2.44]	89.9			Male	0	NA	NA
		Female	11	2.32 [2.11–2.56]	94.1			Female	1	NA	NA
	Inpatient	Total	17	2.05 [1.80–2.34]	97.5		Blank	Total	3	1.88 [1.12–3.13]	92.5
		Male	10	2.44 [2.12–2.80]	87.2			Male	3	1.72 [0.94–3.14]	86.8
		Female	9	2.26 [1.81–2.83]	94.4			Female	3	2.17 [1.40–3.35]	81.0
	Outpatient	Total	2	1.51 [1.11–2.05]	42.7						
		Male	2	1.29 [0.70–2.37]	61.4						
		Female	2	1.74 [1.31–2.31]	0.0						
Representative	Yes	Total	18	2.04 [1.88–2.21]	96.2	Use of structured interview	SI Yes	Total	7	1.63 [1.39–1.92]	58.8
		Male	11	2.25 [2.08–2.44]	89.9			Male	4	2.07 [0.86–5.00]	92.8
		Female	11	2.30 [2.08–2.54]	94.2			Female	3	1.51 [1.15–2.00]	24.1
	No	Total	21	2.01 [1.78–2.27]	96.9		SI No	Total	32	2.08 [1.94–2.24]	97.2
		Male	12	2.31 [2.01–2.64]	86.6			Male	19	2.27 [2.13–2.41]	87.1
		Female	11	2.22 [1.82–2.70]	93.1			Female	19	2.34 [2.14 - 2.55]	94.0
Adjusted by SUD	SUD Yes	Total	4	1.95 [1.41–2.70]	95.0	Adjusted by time-at-risk	Time Yes	Total	7	1.94 [1.60–2.35]	98.8
		Male	2	2.37 [1.71–3.28]	89.8			Male	4	2.37 [2.20–2.54]	78.3
		Female	2	2.62 [2.05–3.37]	85.0			Female	3	2.66 [2.49–2.84]	66
	SUD No	Total	35	2.03 [1.89–2.17]	96.9		Time No	Total	32	2.04 [1.90–2.19]	95.3
		Male	21	2.26 [2.11–2.43]	88.1			Male	19	2.25 [2.05–2.46]	88.0
		Female	20	2.22 [2.02–2.44]	93.9			Female	19	2.19 [1.99–2.42]	92.2

Meta-regression showed no influence in results in proportion of females (*p* = 0.577) and sample size (*p* = 0.228). Sociodemographic variables could not be considered a source of heterogeneity: SDI (*p* = 0.319), HDI (*p* = 0.398) and mid-point year (*p* = 0.578). None of the variables that affect the quality of the studies were significantly related to RR: NOS (*p* = 0.433) and mean follow-up years (*p* = 0.219). When analyzed separately, SMR heterogeneity was 96.7% and HR heterogeneity was 94.4%. Finally, the exclusion of different outcomes did not decrease heterogeneity.

### Level of certainty of the results

Based on the GRADE, the mortality for unnatural causes and suicide showed a high level of certainty, while mortality for all causes and respiratory causes were rated as moderate level of certainty. Evidence for cancer, cardiovascular and cerebrovascular mortality reached a low level of certainty (Table 2). A summary of findings of the GRADE evidence profile can be found as Supplementary Material in Table S6.

## Discussion

The current meta-analysis aimed to systematically review studies regarding mortality by any and specific causes in BD. Results found that individuals with BD have a twofold increased risk of premature mortality when compared to the general population. The largest effect size emerged for suicide, whose risk was 11 times higher in BD, especially in women (17 times higher). Mortality by natural, infectious, respiratory, cardiovascular, and cerebrovascular causes was also elevated in BD. Cancer was the only studied cause with a mortality rate that was not significatively higher in BD.

Over the last years, more attention has been paid to the study of mortality in mental disorders. Although less studied when compared to other disorders (such as schizophrenia and unipolar depression), more evidence has emerged on the increased mortality in individuals with BD [11, 16, 17, 124–126]. Previous studies have assessed mortality in individuals with BD, however a novel and robust systematic review and meta-analysis of mortality in BD was needed, given that the previous reviews did not include the quality assessment, certainty of evidence, rigorous inclusion criteria and a robust assessment of heterogeneity. In contrast to earlier studies, our study provides a detailed analysis about general and specific mortality causes in BD, including not only studies that provided SMR, but a range of studies with other effect measures as RR, HR, MRR, and OR. This approach allows the inclusion of a larger sample, hence better representing the general population of individuals with BD. Our research was able to include 57 studies from 16 different countries (while the meta-analysis from Hayes included 31 studies) and comprised more than twice as many subjects with BD than the previous largest meta-analysis (678 353 vs. 305 859) [17]

In 2020 World Health Organization reported that the leading causes of death in the general population were ischemic heart disease and stroke [127]. In our study, we found an increase of mortality 1.76 and 1.91 times by cardiovascular and cerebrovascular causes in the BD population, respectively. Although different cardiovascular diseases (coronary disease, atherosclerotic disease, arrhythmias, and valvular diseases) can have a different impact on mortality we could not analyze separately the weight of each cause in mortality mainly because the assessment of these causes within the studies were through ICD groups that did not differentiate specific cardiovascular diseases (i.e., I00-I52; I70-I79). Unfortunately, most systems of healthcare are not configured to adequately prevent, diagnose, and treat medical comorbidity in the mood disorder population [128, 129]. Indeed, people with mental disorder do receive lower quality of screening and care for cardiovascular diseases [130].

Risk of natural causes death was almost two times higher in BD, even though natural causes account for most deaths in this population. It is essential that prevention strategies, such as investment in measures to reduce cardiovascular risk factors, begin to be implemented in this population early on. Some ways to reduce these risk factors are through psychoeducation of the target population, encouraging measures that impact adherence to drug treatment, as well as the implementation of programs that engage this population in physical activity, investment in a healthy diet, tobacco cessation, and obesity control. Thus, aiming to better establish functional and effective public health policies, may yield a possible reduction in the impact of modifiable risk factors on mortality in the BD population. Although, it is important to emphasize that this review did not evaluate the weight of risk factors in all and specific-cause mortality in the BD population.

We hypothesize that the increase in respiratory mortality is mainly due to the high index of tobacco use in this population. It is well known that tobacco smoking is 2–3 times higher in bipolar disorder, with estimates ranging from 60–70% in bipolar patients compared to 25–30% in the general population [131]. Unfortunately, the studies included in this meta-analysis did not report rates of smokers, so we could not calculate the weight of tobacco use in the mortality among bipolar disorder individuals. Regarding the increase of infectious mortality in the bipolar disorder population, one possible mechanism is the vulnerability that the lifestyle of the BD population is involved, such as a higher prevalence of substance use, such as alcohol and tobacco, poor diet, and higher rates of sedentarism. These behaviors can negatively impact the immune system response, contributing to a mortality increase in this population.

Cancer was the only cause of death without increase in BD population. Previous metanalyses have shown similar results, however new evidence of increase of mortality by cancer in BD has emerged in the last years [54, 132]. Our main hypothesis is that the factors involved in our findings were related to lower survival in BD subjects than in the general population associated to the fact that cancer is a disease affecting predominantly the late adult or elderly population, and the underdiagnosis of cancer in this population related to disease stigma, lower seeking for medical help, and the difficulties of a proper stage treatment [132, 133]. The lack of significance difference in mortality risk in the analyzed samples might be due to lower screening rates and the risk of missed cancer diagnoses in people with mental disorders compared with the general population. Although, more studies are needed to confirm this hypothesis.

As showed by our data, in agreement with previous literature, the RR for suicide and unnatural causes of death was significative higher in BD than in the general population, namely 11 times increased in BD individuals. Suicide is the leading cause of preventable death [117]. Our findings lead us to an understanding that is necessary an effort to prevent suicide mainly

in the high-risk BD. The improvement of these indices can occur by public health policies implementation through access to information about suicide, training of health professionals to evaluate and manage risk cases, early diagnosis, and implementation of public health policies to prevent suicide [134, 135].

Considering the findings of this study, it is essential to think in how early diagnosis in BD may have an impact on the prevention of disease episodes, clinical comorbidities, adherence to treatment and, consequently, reducing mortality in this group.

Some limitations should be considered. Observational studies in the field of psychiatry are often fraught with several biases (e.g., reverse causation) and also by a poor control of confounders even in large-scale, nationwide, studies [136]. Factors such as disease subtype or symptomatic burden, were not reported in the majority of the included studies. Because of this limitation we were not able to take into account these factors in statistical analysis. Another limitation we could not calculate the mean age of the sample to perform a meta-regression as most of the samples were from observational studies (only eight studies provide mean age), then age was not explored as a source of heterogeneity.

From the statistical point of view, we were not able to find clear sources of heterogeneity based on the classical meta-regression method. Interpreting heterogeneity has been a persistent difficulty in meta-analyses of prevalence studies, which often present high I2 values. Even though, a high I2 value is not always synonymous with high heterogeneity. In meta-analyses of prevalence, I2 statistics may not be discriminative and should be interpreted with caution, avoiding arbitrary thresholds [26].

Prediction intervals predict the range of effect size for any subject randomly assigned from the population in 95% of the time [28] and reflect the variation in treatment effects over different settings, including over future patients [29]. They have been proposed to have a better appreciation of the uncertainty around the effect estimate than CI when heterogeneity is substantial [30] and has been currently recommended in meta-analyses, especially from prevalence studies [27–29]. The prediction Interval of all-cause mortality was 1.37 to 2.98. This interval is entirely above 1 and shows that mortality is elevated when applied in at least 95% of the individual setting. The same rationale can be applied to mortality for suicide (prediction interval 3.71 to 36.90) and respiratory causes (prediction interval 1.65 to 6.12). On the other hand, the prediction interval for cardiovascular (0.87 to 3.58), cerebrovascular (0.91 to 2.70) and cancer (0.67 to 1.46) contain values below 1 and so, although on average the mortality seems to be higher, it may not always be true in an individual setting. In these cases, further research is needed to identify sources of heterogeneity.

The CI higher than one indicates that there is robust evidence to conclude that mortality is higher in most individuals with BD. However, the prediction interval containing it indicates that mortality is heterogeneously distributed, and this result does not apply to 95% of individuals with BD, as CI would suggest. Consequently, there must be factors that hinder mortality for cardiovascular, cerebrovascular, and natural causes to be higher. This rationale has important clinical implications. Now, it is an important topic to explore which characteristics make an individual with BD less susceptible to premature death.

We then conducted meta-regressions to test whether sociodemographic and methodological factors could represent such characteristics. Our study found an I2 of 96.8%, and I2 tells us what proportion of the variance is due to variation in real effects rather than sampling error [27]. It can be interpreted as 3.2% of the differences in mortality are due to sampling or population differences. Hence, it was expected beforehand that meta-regression of sociodemographic characteristics would fail to find differences in heterogeneity. Then, methodological aspects are expected to be relevant. Although not measurable herein, some methodological features can be quoted as potential sources of heterogeneity and further included in studies on mortality in BD, namely: quality of epidemiological data (are databases complete and reliable?), quality of care (could an efficient and accessible health system counterbalance the increased mortality risk in individuals with BD?), and assessment of clinical and psychiatric comorbidities (is there any confounding factor moderating the mortality risk in individuals with BD?).

## Conclusion

According to this meta-analysis, the highest RR for mortality in individuals with BD emerged for suicide, infectious, respiratory, cardiovascular, and cerebrovascular causes. Large-scale and well-designed studies are still needed to identify the main moderators and mediators of premature mortality in the population. Also, efforts must be made to prevent suicide and unnatural mortality causes in the high-risk BD population and to investigate and treat physical comorbidities. Further research should be undertaken to identify modifiable factors that might be targeted by interventions to reduce this gap.

### Supplementary information

Supplemental Material
