# Supplementary material for: All-cause and cause-specific mortality among people with bipolar disorder: a large-scale systematic review and meta-analysis
Source: Mol Psychiatry. 2023 Jul 25;28(6):2508–24. doi: 10.1038/s41380-023-02109-9 (PMC10611575; doi:10.1038/s41380-023-02109-9)
Supplement: Supplementary file 1 — Supplemental Material [file 41380_2023_2109_MOESM1_ESM.docx]

Supplementary material

All-cause and cause-specific mortality among people with bipolar disorder: a large-scale systematic review and meta-analysis

**TABLE S1. SEARCH STRATEGY** **2**

**TABLE S2. EXCLUDED STUDIES AND REASONS FOR EXCLUSION** **3**

**TABLE S3. DETAILED DEMOGRAPHIC CHARACTERISTICS OF INCLUDED STUDIES**…………………………**13**

**TABLE S4. RISK OF BIAS OF THE INCLUDED STUDIES ACCORDING TO THE NEWCASTLE-OTTAWA SCALE (NOS)** **16**

**TABLE S5. GRADE EVIDENCE PROFILE, SUMMARY OF FINDINGS TABLE.** **22**

**TABLE S6. PRISMA Checklist.** **24**

**FIGURE S1. RISK OF BIAS OF INDIVIDUAL STUDIES.** **26**

**FIGURE S2. SUMMARY RISK OF BIAS OF ALL INCLUDED STUDIES.** **27**

**FIGURE S3. PREDICTION INTERVALS AND ITS RESPECTIVE DISTRIBUTION*** **28**

**FIGURE S4. FUNNEL PLOT OF ALL INCLUDED STUDIES IN THE ANALYSIS OF OVERALL MORTALITY** **31**

**REFERENCES** **32**

**Table S1.** Search strategy.

**Database Search terms**

Embase

('bipolar disorder*' OR 'manic-depressive psychos*' OR 'manic depressive psychos*' OR 'manic depressive disorder'/exp OR 'manic depressive disorder' OR 'bipolar affective psychosis' OR 'bipolar affective disorder*'/exp OR 'bipolar affective disorder' OR 'manic disorder*') AND ('mortalit*' OR 'case fatality rate*' OR 'death rate*' OR 'causes of death'/exp OR 'causes of death' OR 'cause of death'/exp OR 'cause of death' OR 'death cause*' OR 'determination of death*' OR 'determinations of death*' OR 'fatal outcome*' OR 'premature death*' OR 'suicide death*') AND [embase]/lim NOT ([embase]/lim AND [medline]/lim)

Retrived references: 1109

PubMed

(Bipolar Disorder[MeSH] OR Bipolar Disorder*[tw] OR Manic-Depressive Psychos*[tw] OR Manic Depressive Psychos*[tw] OR manic depressive disorder[tw] OR Bipolar Affective Psychosis[tw] OR Bipolar affective disorder*[tw] OR Manic Disorder*[tw]) AND (Mortality[MeSH] OR Mortalit*[tw] OR Case Fatality Rate*[tw] OR Death Rate*[tw] OR Causes of Death[tw] OR Cause of Death[tw] OR Death Cause*[tw] OR Determination of Death*[tw] OR Determinations of Death*[tw] OR fatal outcome*[tw] OR Premature Death*[tw] OR suicide death*[tw])

Retrived References: 1170

Web of Science

((Bipolar Disorder* OR Manic-Depressive Psychos* OR Manic Depressive Psychos* OR Bipolar Affective Psychosis OR Manic State* OR Manic Disorder*) AND (Mortalit* OR Case Fatality Rate* OR Death Rate* OR Causes of Death OR Cause of Death OR Death Cause* OR Determination of Death* OR Determinations of Death* OR fatal outcome OR Premature Death* OR suicide death*)

Retrived References: 1965

PsycInfo

((Bipolar Disorder* OR Manic-Depressive Psychos* OR Manic Depressive Psychos* OR Bipolar Affective Psychosis OR Manic State* OR Manic Disorder*) AND (Mortalit* OR Case Fatality Rate* OR Death Rate* OR Causes of Death OR Cause of Death OR Death Cause* OR Determination of Death* OR Determinations of Death* OR fatal outcome OR Premature Death* OR suicide death*)

Retrived References: 1250

| **Table S2.** Excluded studies and reasons for exclusion. | | | |  |
| --- | --- | --- | --- | --- |
| **Study** | **Title** | **Reason for exclusion** | **Observation** | |
| Aagaard 2014 | Valuable intervention against the excess mortality of psychiatric patients | Duplicated sample |  | |
| Aagaard 2016 | Clinically useful predictors for premature mortality among psychiatric patients visiting a psychiatric emergency room | Population | BD not included or not stratified from a broader sample | |
| Abrams 2010 | Preexisting comorbid psychiatric conditions and mortality in nonsurgical intensive care patients. | Study design |  | |
| Abrams 2010 | Influence of psychiatric comorbidity on surgical mortality | Population | BD not included or not stratified from a broader sample | |
| Ajdacic-Gross 2009 | In-patient suicide: A 13-year assessment | Population | BD not included or not stratified from a broader sample | |
| Alharbi 2020 | Trends and predictors of inpatient mortality among patients with mood disorders undergoing PCI | Comparison group | Comparison group with previous somatic diseases | |
| Almeida 2016 | Risk of dementia and death in community-dwelling older men with bipolar disorder | Outcome |  | |
| Almeida 2018 | Substance use among older adults with bipolar disorder varies according to age at first treatment contact | Outcome |  | |
| Almeida 2018 | Older men with bipolar disorder: Clinical associations with early and late onset illness | Outcome |  | |
| Almeida 2019 | Risk of dementia associated with psychotic disorders in later life: the health in men study (HIMS) | Outcome |  | |
| Amaddeo 2007 | Avoidable mortality of psychiatric patients in an area with a community-based system of mental health care | Population | BD not included or not stratified from a broader sample | |
| Antypa 2013 | Clinical, psychological and environmental predictors of prospective suicide events in patients with bipolar disorder | Outcome |  | |
| Arana 2010 | Suicide-related events in patients treated with antiepileptic drugs | Outcome |  | |
| Arffman 2019 | The impact of severe mental illness on lung cancer mortality of patients with lung cancer in Finland in 1990-2013: a register-based cohort study | Population | BD not included or not stratified from a broader sample | |
| Aslan 2020 | Suicidal ideation, behavior, and mortality in male and female US veterans with severe mental illness | Population | Specific subgroup (veterans) | |
| Axelsson 1992 | Factors predicting suicide in psychotic patients | Population | Small sample | |
| Baillargeon 2011 | Effect of mental disorders on diagnosis, treatment, and survival of older adults with colon cancer | Outcome |  | |
| Barcella 2018 | Out-of-hospital cardiac arrest in patients with psychiatric disorder -characteristics and outcomes | Outcome |  | |
| Batty 2012 | Impact of mental health problems on case fatality in male cancer patients | Population | Small sample | |
| Benraad 2020 | Frailty as a predictor of mortality in older adults within 5 years of psychiatric admission | Population | BD not included or not stratified from a broader sample | |
| Beristianos 2013 | Late-life PTSD, comorbid psychiatric and behavioral disorders and risk of mortality among veterans | Population | Specific subgroup (veterans) | |
| Björkenstam 2014 | Suicide in first episode psychosis: A nationwide cohort study | Outcome |  | |
| Black 1998 | Iowa record-linkage study: Death rates in psychiatric patients | Population | BD not included or not stratified from a broader sample | |
| Blackburn 2017 | Statin prescribing for people with severe mental illnesses: a staggered cohort study of 'real-world' impacts | Study design |  | |
| Bodén 2014 | Myocardial infarction survival in patients with bipolar disorder or schizophrenia spectrum disorders-a nationwide cohort study | Duplicated sample |  | |
| Bodén 2015 | Higher mortality after myocardial infarction in patients with severe mental illness: A nationwide cohort study | Comparison group | Comparison group with previous somatic diseases | |

| **Table S2. (cont.)** | | | |  |
| --- | --- | --- | --- | --- |
| **Study** | **Title** | **Reason for exclusion** | **Observation** | |
| Bohnert 2012 | Risk of death from accidental overdose associated with psychiatric and substance use disorders | Outcome |  | |
| Bowersox 2012 | Cause-specific mortality among Veterans with serious mental illness lost to follow-up | Population | Specific subgroup (veterans) | |
| Britton 2017 | Suicide mortality among male veterans discharged from Veterans Health Administration acute psychiatric units from 2005 to 2010 | Population | Specific subgroup (veterans) | |
| Brix 2019 | Death by unnatural causes, mainly suicide, is increased in patients with Hashimoto's thyroiditis. A nationwide Danish register study | Study design |  | |
| Brown 2019 | P58. Patients with psychiatric diagnoses have increased odds of morbidity and mortality in elective orthopedic surgery | Outcome |  | |
| Brugha 2013 | Gender differences in mental health expectancies in early- and midlife in six European countries | Population | BD not included or not stratified from a broader sample | |
| Brunner 2013 | Patients under antidepressants undergoing cardiac surgery have a high risk for adverse events | Comparison group | Comparison group with previous somatic diseases | |
| Bruton 2016 | Assessing the relationship of mood, anxiety, and alcohol related disorders on hospital utilization and mortality in adult patients with sickle cell disease | Outcome |  | |
| Cansiz 2018 | Evaluation of mortality causes among patients with bipolar disorder in a specialized mood clinic | Outcome |  | |
| Carter 2016 | The impact of psychiatric comorbidities on the length of hospital stay in patients with heart failure | Outcome |  | |
| Carter 2016 | The impact of psychiatric comorbidities on the length of hospital stay in patients with heart failure | Outcome |  | |
| Castilho 2020 | Mood disorders and increased risk of non-communicable disease in adults with HIV | Population | BD not included or not stratified from a broader sample | |
| Chander 2010 | Is there earlier time to death among HIV infected individuals with severe mental illness? | Comparison group | Comparison group with previous somatic diseases | |
| Chang 2018 | Fractures and the increased risk of suicide: a population-based case-control study | Population | BD not included or not stratified from a broader sample | |
| Chen 2019 | Adverse stroke outcomes among patients with bipolar disorder | Comparison group | Comparison group with previous somatic diseases | |
| Choi 2020 | Psychiatric disorders and suicide risk among adults with disabilities: A nationwide retrospective cohort study | Population | BD not included or not stratified from a broader sample | |
| Coryell 1999 | Persistence of depressive symptoms and cardiovascular death among patients with affective disorder | Outcome |  | |
| Coryell 2016 | Risk factors for suicide in bipolar i disorder in two prospectively studied cohorts | Study design |  | |
| Crump 2013 | Mental disorders increase the risk of accidental death: A Swedish nationwide cohort study | Population | BD not included or not stratified from a broader sample | |
| Crump 2013 | Mental disorders and vulnerability to homicidal death: Swedish nationwide cohort study | Outcome |  | |
| Cunningham 2015 | Cancer survival in the context of mental illness: A national cohort study | Population | BD not included or not stratified from a broader sample | |
| Das-Munshi 2017 | Race/ethnicity and premature mortality in severe mental illness: cohort study | Population | BD not included or not stratified from a broader sample | |
| Das-Munshi 2017 | Ethnicity and excess mortality in severe mental illness: a cohort study | Population | BD not included or not stratified from a broader sample | |
| Davis 2012 | Reduced Mortality Among Department of Veterans Affairs Patients With Schizophrenia or Bipolar Disorder Lost to Follow-up and Engaged in Active Outreach to Return for Care | Population | BD not included or not stratified from a broader sample | |
| DeHert 2018 | The intriguing relationship between coronary heart disease and mental disorders | Population | BD not included or not stratified from a broader sample | |
| **Table S2. (cont.)** | | | |  |
| **Study** | **Title** | **Reason for exclusion** | **Observation** | |
| Desai 2019 | The prevalence of psychiatric disorders in sudden cardiac arrest survivors: A 5-year nationwide inpatient analysis | Population | BD not included or not stratified from a broader sample | |
| Desai 2020 | Nationwide Frequency, Sequential Trends, and Impact of Co-morbid Mental Health Disorders on Hospitalizations, Outcomes, and Healthcare Resource Utilization in Adult Congenital Heart Disease | Population | BD not included or not stratified from a broader sample | |
| Dickerson 2018 | Natural cause mortality in persons with schizophrenia and bipolar disorder | Population | BD not included or not stratified from a broader sample | |
| Dickerson 2018 | Clinical and Serological Predictors of Suicide in Schizophrenia and Major Mood Disorders | Study design |  | |
| Dickerson 2018 | Natural cause mortality in persons with serious mental illness | Population | BD not included or not stratified from a broader sample | |
| Druss 2011 | Understanding excess mortality in persons with mental illness: 17-year follow up of a nationally representative US survey | Population | BD not included or not stratified from a broader sample | |
| Dutta 2012 | Mortality in first-contact psychosis patients in the U.K.: a cohort study. | Population | BD not included or not stratified from a broader sample | |
| Erlangsen 2017 | Association between spousal suicide and mental, physical, and social health outcomes a longitudinal and nationwide register-based study | Population | BD not included or not stratified from a broader sample | |
| Evans 2018 | Mental health outcomes after major trauma in Ontario: A population-based analysis | Population | BD not included or not stratified from a broader sample | |
| Falsgraf 2017 | Outcomes after traumatic injury in patients with preexisting psychiatric illness | Population | BD not included or not stratified from a broader sample | |
| Fazel 2013 | Premature mortality in epilepsy and the role of psychiatric comorbidity: a total population study | Population | BD not included or not stratified from a broader sample | |
| Fazel 2016 | Mortality, rehospitalization and violent crime in forensic psychiatric patients discharged from hospital: Rates and risk factors | Population | Specific subgroup (forensic psychiatric patients) | |
| Fekadu 2015 | Excess mortality in severe mental illness: 10-Year population-based cohort study rural Ethiopia | Duplicated paper |  | |
| Fernández 2011 | Appropriateness of hospital discharge after a long-term care facility. Comparison between patients discharged at home or at nursing homes | Outcome |  | |
| Fernandez-Quintana 2019 | Suicide and other causes of death among patients admitted to hospital following attempted suicide: a 10-year follow-up study | Population | BD not included or not stratified from a broader sample | |
| Fiedorowicz 2014 | Vascular mortality in participants of the bipolar genomics study | Outcome |  | |
| Fiedorowicz 2014 | Vascular Mortality in Participants of a Bipolar Genomics Study | Duplicated paper |  | |
| Findley 2011 | Excess mortality associated with mental illness and substance use disorders among veteran clinic users with spinal cord injury | Population | Specific subgroup (veterans) | |
| Fond 2019 | Trauma-related mortality of patients with severe psychiatric disorders: population-based study from the French national hospital database | Population | BD not included or not stratified from a broader sample | |
| Fridell 2019 | Prediction of psychiatric comorbidity on premature death in a cohort of patients with substance use disorders: A 42-year follow-up | Population | BD not included or not stratified from a broader sample | |
| Gacouin 2017 | Patients with preexisting psychiatric disorders admitted to ICU: a descriptive and retrospective cohort study | Outcome |  | |
| Gale 2015 | “Association of mental disorders in early adulthood and later psychiatric hospital admissions and mortality in a cohort study of 1 million men”: Correction | Study design |  | |
| Goodwin 2003 | Suicide risk in bipolar disorder during treatment with lithium and divalproex | Population | Included individuals aged 14 years or younger | |
| Grigoriadis 2015 | Perinatal suicide in Ontario: Preliminary results | Population | BD not included or not stratified from a broader sample | |
| **Table S2.** (cont.) | | | |  |
| **Study** | **Title** | **Reason for exclusion** | **Observation** | |
| Guissouma 2020 | Psychiatric profile of patients hospitalized in intensive care unit for suicide attempt | Outcome |  | |
| Haas 2020 | Excess mortality associated with mental illness in people living with HIV in Cape Town, South Africa: a cohort study using linked electronic health records | Population | BD not included or not stratified from a broader sample | |
| Hamel 2018 | Outcomes in renal transplant recipients with bipolar disorder: A large retrospective cohort | Population | Small sample | |
| Hansson 2018 | Risk factors for suicide in bipolar disorder: a cohort study of 12 850 patients | Outcome |  | |
| Hanuš 1996 | Impact of long-term treatment on suicidality in affective disorders | Outcome |  | |
| Harris 2019 | Clinical and Economic Impact of Mental Health Illnesses Surrounding a Gastrointestinal Malignancy Among Elderly Patients | Population | BD not included or not stratified from a broader sample | |
| Himmerich 2019 | Psychiatric comorbidity as a risk factor for mortality in people with anorexia nervosa | Population | BD not included or not stratified from a broader sample | |
| Hiroeh 2001 | Death by homicide, suicide, and other unnatural causes in people with mental illness: A population-based study | Population | BD not included or not stratified from a broader sample | |
| Hiroeh 2008 | Deaths from natural causes in people with mental illness: A cohort study | Population | BD not included or not stratified from a broader sample | |
| Hjerl 2002 | Increased incidence of affective disorders, anxiety disorders, and non-natural mortality in women after breast cancer diagnosis: A nation-wide cohort study in Denmark | Population | BD not included or not stratified from a broader sample | |
| Hjorthøj 2015 | Association between alcohol and substance use disorders and all-cause and cause-specific mortality in schizophrenia, bipolar disorder, and unipolar depression: a nationwide, prospective, register-based study | Duplicated paper |  | |
| Hoang 2011 | Mortality after hospital discharge for people with schizophrenia or bipolar disorder: Retrospective study of linked English hospital episode statistics, 1999-2006 | Duplicated paper |  | |
| Hoertel 2014 | Poor longitudinal continuity of care is associated with an increased mortality rate among patients with mental disorders: results from the French National Health Insurance Reimbursement Database | Outcome |  | |
| Honkonen 2008 | Mortality of Finnish acute psychiatric hospital patients | Population | BD not included or not stratified from a broader sample | |
| Horsdal 2017 | C-reactive protein and white blood cell levels in schizophrenia, bipolar disorders and depression - associations with mortality and psychiatric outcomes: a population-based study | Study design |  | |
| Hossain 2019 | Medical and Psychiatric Comorbidities in Bipolar Disorder: Insights from National Inpatient Population-based Study | Study design |  | |
| Iglay 2017 | Impact of preexisting mental illness on all-cause and breast cancer-specific mortality in elderly patients with breast cancer | Population | BD not included or not stratified from a broader sample | |
| Ilgen 2010 | "Violent and nonviolent suicide in veterans with substance-use disorders": Erratum | Study design |  | |
| Ilgen 2010 | Violent and nonviolent suicide in veterans with substance-use disorders | Population | Specific subgroup (veterans) | |
| Ilgen 2010 | Psychiatric diagnoses and risk of suicide in veterans | Population | Specific subgroup (veterans) | |
| Ilgen 2012 | Psychopathology, Iraq and Afghanistan service, and suicide among Veterans Health Administration patients | Population | Specific subgroup (veterans) | |
| Innes 1970 | Mortality among psychiatric patients. | Population | BD not included or not stratified from a broader sample | |
| Ishida 2019 | Lower proportion of fatal arrhythmia in sudden cardiac arrest among patients with severe mental illness than nonpsychiatric patients | Population | BD not included or not stratified from a broader sample | |
| Ishida 2020 | Lower proportion of fatal arrhythmia in sudden cardiac arrest among patients with severe mental illness than nonpsychiatric patients | Population | BD not included or not stratified from a broader sample | |
| **Table S2.** (cont.). | | | |  |
| **Study** | **Title** | **Reason for exclusion** | **Observation** | |
| Isometsä 1994 | Suicide in bipolar disorder in Finland | Study design |  | |
| John 2018 | Premature mortality among people with severe mental illness — New evidence from linked primary care data | Population | BD not included or not stratified from a broader sample | |
| John 2020 | Mental disorders and total mortality after 20 years in an adult general population sample | Population | Small sample | |
| Jokinen 2009 | HPA axis hyperactivity and cardiovascular mortality in mood disorder inpatients | Outcome |  | |
| Jonsson 2013 | Diagnosis-specific disability pension predicts suicidal behavior and mortality in young adults: A nationwide prospective cohort study | Population | BD not included or not stratified from a broader sample | |
| Jørgensen 1992 | Cause of death in reactive psychosis | Outcome |  | |
| Juurlink 2004 | Medical illness and the risk of suicide in the elderly | Population | BD not included or not stratified from a broader sample | |
| Keinanen 2018 | Mortality in people with psychotic disorders in Finland: A population-based 13-year follow-up study | Population | BD not included or not stratified from a broader sample | |
| Kessing 2015 | Life expectancy in bipolar disorder | Outcome |  | |
| Khalsa 2008 | Suicidal events and accidents in 216 first-episode bipolar I disorder patients: Predictive factors | Study design |  | |
| Kilbourne 2009 | Excess heart-disease-related mortality in a national study of patients with mental disorders: identifying modifiable risk factors | Population | Specific subgroup (veterans) | |
| Kim 2017 | Mortality in Schizophrenia and Other Psychoses: Data from the South Korea National Health Insurance Cohort, 2002-2013 | Population | BD not included or not stratified from a broader sample | |
| Kimmel 2019 | Psychiatric Illness and Mortality in Hospitalized ESKD Dialysis Patients | Population | BD not included or not stratified from a broader sample | |
| Kisely 2012 | The effect of community treatment orders on mortality: A population-based study from Western Australia | Population | BD not included or not stratified from a broader sample | |
| Kisely 2016 | Why do psychiatric patients have higher cancer mortality rates when cancer incidence is the same or lower? | Population | BD not included or not stratified from a broader sample | |
| Krupchanka 2018 | Mortality in people with mental disorders in the Czech Republic: a nationwide, register-based cohort study | Population | BD not included or not stratified from a broader sample | |
| Kugathasan 2019 | Coronary artery calcification and mortality risk in patients with severe mental illness: a retrospective multicenter cohort study | Population | BD not included or not stratified from a broader sample | |
| Lähteenvuo 2019 | Suicide mortality and use of psychotropic drugs in patients hospitalized due to bipolar disorder: A Finnish nationwide cohort study | Outcome |  | |
| Laursen 2011 | Heart disease treatment and mortality in schizophrenia and bipolar disorder - Changes in the danish population between 1994 and 2006 | Outcome |  | |
| Laursen 2011 | Life expectancy among persons with schizophrenia or bipolar affective disorder | Outcome |  | |
| Laursen 2013 | Life expectancy and cardiovascular deaths of patients with bipolar disorder or schizophrenia in the Nordic countries | Duplicated sample |  | |
| Laursen 2014 | Cardiovascular drug use and mortality in patients with schizophrenia or bipolar disorder: a Danish population-based study | Comparison group | Comparison group with previous somatic diseases | |
| Lavender 2016 | Violent deaths among Georgia workers: An examination of suicides and homicides by occupation, 2006–2009 | Population | BD not included or not stratified from a broader sample | |
| Lawrence 2000 | Suicide and attempted suicide among older adults in Western Australia | Population | BD not included or not stratified from a broader sample | |
| Lee 2020 | Impact of Onset of Psychiatric Disorders and Psychiatric Treatment on Mortality Among Patients with Cancer | Population | BD not included or not stratified from a broader sample | |
| **Table S2.** (cont.) | | | |  |
| **Study** | **Title** | **Reason for exclusion** | **Observation** | |
| Leece 2015 | Predictors of Opioid-Related Death During Methadone Therapy | Population | BD not included or not stratified from a broader sample | |
| Lemogne 2013 | Mortality associated with depression as compared with other severe mental disorders: A 20-year follow-up study of the GAZEL cohort. | Population | BD not included or not stratified from a broader sample | |
| Leray 2013 | Suicide in multiple sclerosis: Insights from the SURVIMUS study, a large multicentre study on long-term mortality of French patients | Population | BD not included or not stratified from a broader sample | |
| Lloyd 2019 | Mental health disorders are more common in colorectal cancer survivors and associated with decreased overall survival | Outcome |  | |
| Löfman 2017 | Affective disorders and completed suicide by self-poisoning, trend of using antidepressants as a method of self-poisoning | Outcome |  | |
| Lu 2010 | Using multiple-cause-of-death data as a complement of underlying-cause-of-death data in examining mortality differences in psychiatric disorders between countries. | Population | BD not included or not stratified from a broader sample | |
| Mai 2011 | Mental illness related disparities in diabetes prevalence, quality of care and outcomes: A population-based longitudinal study | Outcome |  | |
| Mansuri 2016 | Trends of hospitalization for bipolar I in USA: A nationwide analysis | Outcome |  | |
| Martin 1985 | Mortality in a follow-up of 500 psychiatric outpatients: I. Total mortality | Population | Small sample | |
| Mattisson 2015 | Mortality of subjects with mood disorders in the Lundby community cohort: A follow-up over 50 years | Population | BD not included or not stratified from a broader sample | |
| McCarthy 2013 | Suicide Mortality Following Nursing Home Discharge in the Department of Veterans Affairs Health System | Population | Specific subgroup (veterans) | |
| Medici 2015 | Secular Trend in Incidence and Mortality of Bipolar Disorder | Duplicated paper |  | |
| Merrill 2014 | Drug combinations contributing to death according to mental health status | Study design |  | |
| Mitrović 2019 | Discrepancies between clinical and autopsy diagnosis of cause of death among psychiatric patients who died due to natural causes. A retrospective autopsy study | Outcome |  | |
| Moalla 2015 | Alcohol abuse, impulsivity and suicide attempts in bipolar disorders | Outcome |  | |
| Molnar 2018 | History of psychosis and mania, and outcomes after kidney transplantation - a retrospective study | Outcome |  | |
| Molnar 2018 | Psychotic disorders and outcomes after kidney transplantation | Population | BD not included or not stratified from a broader sample | |
| Montes 2009 | Cardiovascular risk in a Spanish population of bipolar disorder patients: Results from the BIMET study | Outcome |  | |
| Muller-Oerlinghausen 1992 | The effect of long-term lithium treatment on the mortality of patients with manic-depressive and schizoaffective illness | Outcome |  | |
| MullerOerlinghausen 1996 | Mortality of patients who dropped out from regular lithium prophylaxis: A collaborative study by the International Group for the Study of Lithium-Treated Patients (IGSLI) | Outcome |  | |
| Nakajo 2014 | Acute mortality among psychiatric patients with acute agitation using a Japanese hospital database | Population | Specific subgroup (patients with acute agitation) | |
| Nayeri 2017 | Pre-existing psychiatric illness is associated with increased risk of recurrent takotsubo cardiomyopathy | Population | BD not included or not stratified from a broader sample | |
| Nielsen 2011 | Psychiatric disorders and mortality among people in homeless shelters in Denmark: A nationwide register-based cohort study | Population | Specific subgroup (people in homeless shelters) | |
| Nilsson 1995 | Mortality in recurrent mood disorders during periods on and off lithium a complete population study in 362 patients | Population | BD not included or not stratified from a broader sample | |

| **Table S2.** (cont.) | | | |  |
| --- | --- | --- | --- | --- |
| **Study** | **Title** | **Reason for exclusion** | **Observation** | |
| Nordentoft 2011 | Absolute Risk of Suicide After First Hospital Contact in Mental Disorder | Outcome |  | |
| Nordstrom 1995 | Attempted suicide predicts suicide risk in mood disorders | Outcome |  | |
| Olfson 2016 | Short-Term suicide risk after psychiatric hospital discharge | Outcome |  | |
| Ostergaard 2017 | Associations between substance use disorders and suicide or suicide attempts in people with mental illness: a Danish nation-wide, prospective, register-based study of patients diagnosed with schizophrenia, bipolar disorder, unipolar depression or personal | Outcome |  | |
| Pan 2016 | Three-year mortality in relation to early hospitalization and number of outpatient clinic visits in people with newly diagnosed bipolar disorder | Outcome |  | |
| Pan 2017 | Transformation of excess mortality in people with schizophrenia and bipolar disorder in Taiwan | Duplicated paper |  | |
| Parisi 2017 | Psychiatric comorbidity, psychotropic medication prescribing and suicidality in patients with psoriasis: A population-based cohort study | Population | BD not included or not stratified from a broader sample | |
| Park 2008 | Effect of sociodemographic factors, cancer, psychiatric disorder on suicide: gender and age-specific patterns | Outcome |  | |
| Park 2013 | Suicide mortality and risk factors in the 12 months after discharge from psychiatric inpatient care in Korea: 1989-2006 | Outcome |  | |
| Pinto 2012 | Factors associated with suicide mortality among the elderly in Brazilian municipalities between 2005 and 2007 | Population | BD not included or not stratified from a broader sample | |
| Pirkola 2007 | Reductions in post discharge suicide after deinstitutionalization and decentralization: A nationwide register study in Finland | Population | BD not included or not stratified from a broader sample | |
| Plana-Ripoll 2019 | A comprehensive analysis of mortality-related health metrics associated with mental disorders: a nationwide, register-based cohort study | Population | BD not included or not stratified from a broader sample | |
| Popiolek 2018 | Rehospitalization and suicide following electroconvulsive therapy for bipolar depression–A population-based register study | Population | Specific subgroup (bipolar depression after ECT) | |
| Postolache 2010 | Seasonal spring peaks of suicide in victims with and without prior history of hospitalization for mood disorders | Population | BD not included or not stratified from a broader sample | |
| Prieto 2015 | Risk of myocardial infarction and stroke in bipolar disorder: A population-based cohort study | Outcome |  | |
| Prieto 2016 | Long-term risk of myocardial infarction and stroke in bipolar i disorder: A population-based Cohort Study | Outcome |  | |
| Prisciandaro 2011 | Impact of psychiatric comorbidity on mortality in veterans with type 2 diabetes | Population | Specific subgroup (veterans) | |
| Protty 2017 | Increased morbidity, mortality and length of in-hospital stay for patients with acute coronary syndrome with premorbid psychiatric diagnoses | Population | BD not included or not stratified from a broader sample | |
| Puri 1995 | Mortality in a hospitalized mentally handicapped population: A 10-year survey | Population | Specific subgroup (hospitalized mentally handicapped population) | |
| Ramsey 2012 | Lifetime manic spectrum syndromes and all-cause mortality: A26-year follow-up of the us national epidemiological catchment area study | Duplicated sample |  | |
| Räsänen 2003 | Excess mortality among long-stay psychiatric patients in Northern Finland | Population | Specific subgroup (long-stay psychiatric patients) | |
| Reilly 2009 | Renal outcomes in people with bipolar disorder treated with lithium: A retrospective cohort database study | Outcome |  | |
| Reutfors 2010 | Suicide and hospitalization for mental disorders in Sweden: A population-based case-control study | Population | BD not included or not stratified from a broader sample | |

| **Table S2.** (cont.) | | | |  |
| --- | --- | --- | --- | --- |
| **Study** | **Title** | **Reason for exclusion** | **Observation** | |
| Ribe 2014 | Long-term mortality of persons with severe mental illness and diabetes: a population-based cohort study in Denmark | Population | BD not included or not stratified from a broader sample | |
| Ribe 2015 | Thirty-day mortality after infection among persons with severe mental illness: a population-based cohort study in Denmark | Population | BD not included or not stratified from a broader sample | |
| Risgaard 2015 | Sudden cardiac death in young adults with previous hospital-based psychiatric inpatient and outpatient treatment: a nationwide cohort study from Denmark | Population | BD not included or not stratified from a broader sample | |
| Rockett 2007 | Suicide-associated comorbidity among US males and females: a multiple cause-of-death analysis | Population | BD not included or not stratified from a broader sample | |
| Rockett 2009 | Discrepant comorbidity between minority and white suicides: A national multiple cause-of-death analysis | Population | BD not included or not stratified from a broader sample | |
| Roy 1995 | Suicide among psychiatric hospital in-patients | Population | Small sample | |
| Schaffer 2012 | Suicide by overdose in a bipolar disorder cohort | Outcome |  | |
| Schaffer 2013 | Suicide deaths in bipolar disorder: Sex-based differences in demographic, clinical and suicide-specific factors | Outcome |  | |
| Schaffer 2015 | Rates of suicide death in people with bipolar disorder: Findings from the ISBD Task Force on Suicide | Study design |  | |
| Schaffer 2015 | Epidemiology, neurobiology and pharmacological interventions related to suicide deaths and suicide attempts in bipolar disorder: Part I of a report of the International Society for Bipolar Disorders Task Force on Suicide in Bipolar Disorder | Study design |  | |
| Schulman-Marcus 2016 | Disparities in revascularization for ST-elevation myocardial infarction persist for patients with severe mental illness | Comparison groupp | Comparison group with previous somatic diseases | |
| Shalaby 2012 | Mood disorders and outcome in patients receiving cardiac resynchronization therapy | Population | BD not included or not stratified from a broader sample | |
| Simon 2007 | Risk of suicide attempt and suicide death in patients treated for bipolar disorder | Duplicated paper |  | |
| Simon 2018 | Mortality rates after the first diagnosis of psychotic disorder in adolescents and young adults | Population | BD not included or not stratified from a broader sample | |
| Singhal 2014 | Risk of self-harm and suicide in people with specific psychiatric and physical disorders: comparisons between disorders using English national record linkage | Outcome |  | |
| StaudtHansen 2018 | Increasing mortality gap for patients diagnosed with bipolar disorder—a nationwide study with 20 years of follow‐up | Duplicated sample |  | |
| StaudtHansen 2019 | Increasing mortality gap for patients diagnosed with bipolar disorder—A nationwide study with 20 years of follow-up | Duplicated paper |  | |
| Steingrímsson 2016 | Total population-based study of the impact of substance use disorders on the overall survival of psychiatric inpatients | Population | BD not included or not stratified from a broader sample | |
| Strauss 2020 | Mood Disorders in Late Life: A Population-based Analysis of Prevalence, Risk Factors, and Consequences in Community-dwelling Older Adults in Ontario | Population | BD not included or not stratified from a broader sample | |
| Strudsholm 2005 | Increased risk for pulmonary embolism in patients with bipolar disorder | Outcome |  | |
| Sutaria 2016 | Trends of hospitalization for major bipolar I (most recent episode-manic) in USA: A nationwide analysis | Outcome |  | |
| Suvisaari 2013 | Mortality and its determinants in people with psychotic disorder | Population | BD not included or not stratified from a broader sample | |
| Takeda 2019 | Mortality and suicide rates in patients discharged from forensic psychiatric wards in Japan | Population | BD not included or not stratified from a broader sample | |
| Tidemalm 2008 | Risk of suicide after suicide attempt according to coexisting psychiatric disorder: Swedish cohort study with long term follow-up | Population | BD not included or not stratified from a broader sample | |

| **Table S2.** (cont.) | | | | |  |  |
| --- | --- | --- | --- | --- | --- | --- |
| **Study** | **Title** | | **Reason for exclusion** | **Observation** | |  |
| Toender 2018 | Impact of severe mental illness on cancer stage at diagnosis and subsequent mortality: A population-based register study | | Population | BD not included or not stratified from a broader sample | |  |
| Toffol 2015 | Lithium is associated with decrease in all-cause and suicide mortality in high-risk bipolar patients: A nationwide registry-based prospective cohort study | | Outcome |  | |  |
| Tong 2010 | Cohort-specific risk of suicide for different mental disorders in China | | Population | BD not included or not stratified from a broader sample | |  |
| Townsend 2017 | The prevalence of psychiatric diagnoses and associated mortality in hospitalized US trauma patients | | Population | BD not included or not stratified from a broader sample | |  |
| Trivedi 2017 | Preliminary analysis of life expectancy and common causes of death among veterans with mental illnesses | | Population | Specific subgroup (veterans) | |  |
| Tsai 2005 | A retrospective analysis of risk and protective factors for natural death in bipolar disorder | | Outcome |  | |  |
| Tsai 2011 | Risk and protective factors for premature circulatory mortality of bipolar disorder | | Comparison group | Comparison group with previous somatic diseases | |  |
| Tsuang 1977 | Mortality in patients with schizophrenia, mania, depression and surgical conditions | | Population | BD not included or not stratified from a broader sample | |  |
| Tsuang 1977 | Mortality in Patients with Schizophrenia, Mania, Depression and Surgical Conditions: A Comparison with General Population Mortality | | Duplicated sample |  | |  |
| Tsuang 1978 | Excess Mortality in Schizophrenia and Affective Disorders: Do Suicides and Accidental Deaths Solely Account for This Excess? | | Duplicated sample |  | |  |
| Tsuang 1978 | Suicide in schizophrenics, manics, depressives, and surgical controls: A comparison with general population suicide mortality | | Duplicated sample |  | |  |
| Tsuang 1980 | Premature deaths in schizophrenia and affective disorders: An analysis of survival curves and variables affecting a shortened survival | | Population | BD not included or not stratified from a broader sample | |  |
| Vance 2019 | Increased cardiovascular disease risk in veterans with mental illness | | Population | Specific subgroup (veterans) | |  |
| Vestergaard 1991 | Five-year mortality in lithium-treated manic-depressive patients | | Population | BD not included or not stratified from a broader sample | |  |
| Wang 2020 | Identification of psychiatric patients with high mortality and low medical utilization: a population-based propensity score-matched analysis | | Population | BD not included or not stratified from a broader sample | |  |
| Weeke 1987 | Cardiovascular death and manic-depressive psychosis | | Duplicated paper |  | |  |
| Weiner 2011 | Cardiovascular morbidity and mortality in bipolar disorder | | Study design |  | |  |
| Weng 2016 | Factors influencing attempted and completed suicide in postnatal women: A population-based study in Taiwan | | Population | BD not included or not stratified from a broader sample | |  |
| Wilson 2019 | Place of death and other factors associated with unnatural mortality in patients with serious mental disorders: population-based retrospective cohort study | | Population | BD not included or not stratified from a broader sample | |  |
| Windfuhr 2011 | The incidence and associated risk factors for sudden unexplained death in psychiatric in-patients in England and Wales | | Population | BD not included or not stratified from a broader sample | |  |
| Wong 2019 | Incidence of hepatocellular carcinoma and cirrhotic complications in patients with psychiatric illness: A territory-wide cohort study | | Population | BD not included or not stratified from a broader sample | |  |
| Wu 2013 | The Incidence and Relative Risk of Stroke among Patients with Bipolar Disorder: A Seven-Year Follow-Up Study | | Comparison group | Comparison group with previous somatic diseases | |  |
| Yates 1987 | Cardiovascular risk factors in affective disorder | | Outcome |  | |  |
| Yip 2020 | High incidence of hepatocellular carcinoma and cirrhotic complications in patients with psychiatric illness: A territory-wide cohort study | | Population | BD not included or not stratified from a broader sample | |  |
| **Table S2.** (cont.) | | | | |  | |
| **Study** | **Title** | **Reason for exclusion** | | **Observation** | | |
| Yoon 2011 | Effect of comorbid alcohol and drug use disorders on premature death among unipolar and bipolar disorder decedents in the United States, 1999 to 2006 | Outcome | |  | | |
| Yue 2017 | Associations between severe mental illness and healthcare expenditure after acute myocardial infarction: A nationwide retrospective cohort study | Population | | BD not included or not stratified from a broader sample | | |
| Zgueb 2014 | Deaths in a Tunisian psychiatric hospital: An eleven-year retrospective study | Population | | Specific subgroup (deaths in a Tunisian psychiatric hospital) | | |
| Zhu 2017 | First-onset mental disorders after cancer diagnosis and cancer-specific mortality: a nationwide cohort study | Population | | BD not included or not stratified from a broader sample | | |
| Zubenko 1997 | Mortality of elderly patients with psychiatric disorders | Population | | BD not included or not stratified from a broader sample | | |
| Mayer Jr 2019 | Mood disorders impaired quality of life but not the mortality or morbidity risk in stable coronary heart disease patients | Outcome | |  | | |

**Table S3.** Detailed demographic characteristics of included studies.

|  | **SDI** | **HDI** | **Specificities** | **Study Design** | **Adjusted by** |
| --- | --- | --- | --- | --- | --- |
| Ahrens 19951 | NA | NA | BD receiving lithium | Retrospective | Age, gender |
| Ajetunmobi 20132 | 0.85 | 0.932 | No | Retrospective | Age, gender, time at risk, deprivation index |
| Almeida 20163 | 0.84 | 0.944 | No | Prospective | Age, gender |
| Angst 20024 | 0.93 | 0.955 | No | Prospective | Age, gender, time at risk |
| Angst 20055 | 0.93 | 0.955 | No | Prospective | Age, gender, time at risk |
| Angst 20136 | 0.93 | 0.955 | No | Prospective | Age, gender, time at risk |
| Black 19877 | 0.86 | 0.926 | No | Retrospective | Age, gender |
| Bratfos 19688 | 0.91 | 0.957 | No | Prospective | Age, gender |
| Callaghan 20149 | 0.86 | 0.926 | No | Prospective | Age, gender, ethnic group |
| Castagnini 201310 | 0.89 | 0.940 | No | Prospective | Age, gender |
| Chang 201011 | 0.85 | 0.932 | No | Prospective | Age, gender, ethnic group |
| Chang 201212 | 0.85 | 0.932 | No | Prospective | Age, gender, ethnic group |
| Chen 201013 | 0.87 | 0.911 | No | Case-control | Age, gender, psychiatric comorbidity |
| Chen 202014 | 0.87 | 0.911 | No | Retrospective | Age, gender |
| Choi 201915 | 0.88 | 0.916 | No | Case-control | Age, gender, income, deprivation index, urbanization |
| Crump 201316 | 0.87 | 0.945 | No | Retrospective | Age, gender, marital status, educational level, employment status, income, SUD |
| Dutta 200717 | 0.85 | 0.932 | No | Retrospective | Age, gender |
| Fekadu 201518 | 0.08 | 0.485 | No | Prospective | Age, gender |
| Fiedorowicz 200919 | 0.86 | 0.926 | No | Prospective | Age, gender |
| Gale 201220 | 0.87 | 0.945 | No | Retrospective | Age, gender, educational level, income, medical comorbidity |
| Guan 201321 | 0.88 | 0.944 | No | Retrospective | Age, gender, income |
| Hayes 201722 | 0.85 | 0.932 | No | Retrospective | Age, gender, deprivation index, medical comorbidity |
| Hjorthøj 201523 | 0.89 | 0.940 | No | Prospective | Age, gender, SUD |
| Hoang 201124 | 0.85 | 0.932 | No | Retrospective | Age, gender |
| Hoang 201325 | 0.85 | 0.932 | No | Prospective | Age, gender |
| Høye 201626 | 0.91 | 0.957 | No | Prospective | Age, gender |

| **Table S3.** (cont.) |  |  |  |  |  |
| --- | --- | --- | --- | --- | --- |
|  | **SDI** | **HDI** | **Specificities** | **Study Design** | **Adjusted by** |
| Kay 197727 | 0.87 | 0.945 | No | Prospective | Age, gender |
| Kim 201828 | 0.88 | 0.916 | No | Retrospective | Age, gender, income, urbanization, medical comorbidity |
| Kodesh 201229 | 0.8 | 0.919 | No | Retrospective | Age, gender |
| Laursen 200730 | 0.87 | 0.945 | No | Retrospective | Age, gender |
| Laursen 200931 | 0.89 | 0.940 | BD with heart diseases | Prospective | Age, gender |
| Laursen 201132 | 0.89 | 0.940 | No | Retrospective | Age, gender |
| Laursen 2013a33 | 0.89 | 0.940 | No | Prospective | Age, gender |
| Laursen 2013b34 | 0.86 | 0.938 | No | Prospective | Age, gender |
| Laursen 2013c34 | 0.87 | 0.945 | No | Prospective | Age, gender |
| Lomholt 201935 | 0.89 | 0.940 | No | Prospective | Age, gender |
| Medici 201536 | 0.89 | 0.940 | No | Prospective | Gender |
| Mohamed 201937 | 0.86 | 0.926 | BD with MI | Retrospective | Gender |
| Newman 199138 | 0.87 | 0.929 | No | Retrospective | Age, gender |
| Norton 198439 | 0.85 | 0.932 | BD receiving lithium | Retrospective | Age, gender |
| Osborn 2007/200840,41 | 0.85 | 0.932 | No | Retrospective | Age, gender, deprivation index |
| Ösby 200142 | 0.87 | 0.945 | No | Retrospective | Age, gender |
| Pan 201743 | 0.87 | 0.911 | No | Retrospective | Age, gender, time at risk |
| Pan 202044 | 0.87 | 0.911 | No | Retrospective | Age, gender, time at risk |
| Park 201945 | 0.88 | 0.916 | No | Retrospective | Age, gender |
| Ramsey 201346 | 0.86 | 0.926 | No | Retrospective | Age, gender, marital status, educational level |
| Saku 199547 | 0.87 | 0.919 | No | Prospective | Age, time at risk |
| Schaffer 201448 | 0.87 | 0.929 | No | Retrospective | Age, gender, marital status, income, medical comorbidity |
| Schneider 200149 | 0.9 | 0.947 | No | Prospective | Age, gender |
| Schulman-Marcus 201650 | 0.86 | 0.926 | No | Retrospective | Age, gender, income, psychiatric comorbidity, medical comorbidity |
| Sharma 199451 | 0.85 | 0.932 | No | Prospective | Age, gender |

| **Table S3.** (cont.) |  |  |  |  |  |
| --- | --- | --- | --- | --- | --- |
|  | **SDI** | **HDI** | **Specificities** | **Study Design** | **Adjusted by** |
| Tsuang 198052 | 0.86 | 0.926 | No | Prospective | Age, gender |
| Vinogradova 201053 | 0.85 | 0.932 | BD with diabetes | Prospective | Age, gender, deprivation index |
| Webb 201454 | 0.87 | 0.945 | No | Retrospective | Age, gender, marital status, income, psychiatric comorbidity |
| Weeke 198755 | 0.89 | 0.940 | No | Retrospective | Age, gender, time at risk |
| Westman 201356 | 0.87 | 0.945 | No | Retrospective | Age, gender |
| Yeh 201957 | 0.86 | 0.926 | No | Case-control | Age, gender, psychiatric comorbidity |

BD: bipolar disorders; HDI: Human Development Index; MI, myocardial infarction; NA, not available; SDI, Social Development Index; SUD, substance use disorder;

**Table S4**. Risk of bias of the included studies according to the Newcastle-Ottawa Scale (NOS)

**NOS Section NOS Scoring Authors` notes**

1. Representativeness of the exposed cohort
2. Selection of the non-exposed cohort
3. Ascertainment of exposure
4. Demonstration that outcome of interest was
5. truly representative * Truly representative of the average individuals with bd in the community
6. somewhat representative * Somewhat representative of the average individuals with bd in the community *
7. selected group of users Only inpatients - after psychiatric hospitalization, only lithium users, only substance use disorder
8. no description of the derivation of the cohort
9. drawn from the same community as the exposed cohort *
10. drawn from a different source
11. no description of the derivation of the non-exposed cohort
12. secure record (eg., surgical records) *
13. structured interview *
14. written self-report
15. no description
16. yes *

In the case of mortality studies, the outcome of interest is still the presence of a disease/ incident, rather than

not present at start of study

1. Comparability of cohorts
2. Assessment of outcome
3. no
4. study controls for AGE *
5. study controls for GENDER *
6. independent blind assessment *
7. record linkage *
8. self-report
9. no description

death. That is to say that a statement of no history of disease or incident earns a star. In all studies, bipolar disorder was obviously presented from the beginning

1. Was follow-up long enough for outcomes to occur?
2. Adequacy of follow up of cohorts
3. yes * At least a 5-years follow-up
4. no
5. complete follow-up; all subjects accounted for *
6. subjects lost to follow-up unlikely to introduce bias * Small number lost - > 95% follow up, or description provided of those lost
7. follow up rate < 95% and no description of those lost
8. no statement

* Such item adds point to the total score.

**Table S4.** Risk of bias of the included studies according to the Newcastle-Ottawa Scale (NOS)

| **Section** | | **Ahrens 1995** | **Ajetunmobi 2013** | **Almeida 2016** | **Angst 2002** | **Angst 2005** | **Angst 2013** | **Black 1987** | **Bratfos 1968** | **Callaghan 2014** | **Castagnini 2013** | **Chang 2010** |
| --- | --- | --- | --- | --- | --- | --- | --- | --- | --- | --- | --- | --- |
| 1 | a |  |  |  |  |  |  |  |  |  |  |  |
|  | b | (Multicentric, small sample) | (Large sample) | (Community, small sample) |  |  |  |  |  | (Large sample) |  | (Community, small sample) |
|  | c |  |  |  | (Small sample) | (Small sample) | (Small sample) | (Small sample) | (Small sample) |  | (Small sample) |  |
|  | d |  |  |  |  |  |  |  |  |  |  |  |
| 2 | a |  |  |  |  |  |  |  |  |  |  |  |
|  | b |  |  |  |  |  |  |  |  |  |  |  |
|  | c |  |  |  |  |  |  |  |  |  |  |  |
| 3 | a |  |  |  |  |  |  |  |  |  |  |  |
|  | b |  |  |  |  |  |  |  |  |  |  |  |
|  | c |  |  |  |  |  |  |  |  |  |  |  |
|  | d |  |  |  |  |  |  |  |  |  |  |  |
| 4 | a |  |  |  |  |  |  |  |  |  |  |  |
|  | b |  |  |  |  |  |  |  |  |  |  |  |
| 5 | a |  |  |  |  |  |  |  |  |  |  |  |
|  | b |  |  |  |  |  |  |  |  |  |  |  |
| 6 | a |  |  |  |  |  |  |  |  |  |  |  |
|  | b |  |  |  |  |  |  |  |  |  |  |  |
|  | c |  |  |  |  |  |  |  |  |  |  |  |
|  | d |  |  |  |  |  |  |  |  |  |  |  |
| 7 | a |  | (10 years) | (12.7 years) | (34–38 years) | (40-44 years) | (48-52 years) | (2-14 years) | (10 years) | (7.6 years) | (6.6 years) |  |
|  | b | (6 months) |  |  |  |  |  |  |  |  |  | (3 years) |
| 8 | a |  |  |  |  |  |  |  |  |  |  |  |
|  | b |  |  |  | (99.3%) |  | (99.3%) |  |  |  |  |  |
|  | c | (394 dropouts) |  |  |  |  |  |  | (81.0%) |  |  |  |
|  | d |  |  |  |  |  |  |  |  |  |  |  |
| **Score** | | **5** | **7** | **8** | **7** | **6** | **7** | **6** | **6** | **7** | **6** | **7** |

**Table S4.** (cont.)

| **Section** | | **Chang 2012** | **Chen 2010** | **Chen 2020** | **Choi 2019** | **Crump 2013** | **Dutta 2007** | **Fekadu 2015** | **Fiedorowicz 2009** | **Gale 2012** | **Guan 2013** | **Hayes 2017** |
| --- | --- | --- | --- | --- | --- | --- | --- | --- | --- | --- | --- | --- |
| 1 | a |  |  | (Community, large sample) | (Community, large sample) | (Community, large sample) |  |  |  | (Community, large sample) | (Community, large sample) | (Community, large sample) |
|  | b |  | (Community, small sample) |  |  |  |  | (Community, small sample) | (Community, small sample) |  |  |  |
|  | c | (Large sample, |  |  |  |  | (Only type I BD, small sample) |  |  |  |  |  |
|  |  | only Depression) |  |  |  |  |  |  |  |  |  |  |
|  | d |  |  |  |  |  |  |  |  |  |  |  |
| 2 | a |  |  |  |  |  |  |  |  |  |  |  |
|  | b |  |  |  |  |  |  |  |  |  |  |  |
|  | c |  |  |  |  |  |  |  |  |  |  |  |
| 3 | a |  |  |  |  |  |  |  |  |  |  |  |
|  | b |  |  |  |  |  |  |  |  |  |  |  |
|  | c |  |  |  |  |  |  |  |  |  |  |  |
|  | d |  |  |  |  |  |  |  |  |  |  |  |
| 4 | a |  |  |  |  |  |  |  |  |  |  |  |
|  | b |  |  |  |  |  |  |  |  |  |  |  |
| 5 | a |  |  |  |  |  |  |  |  |  |  |  |
|  | b |  |  |  |  |  |  |  |  |  |  |  |
| 6 | a |  |  |  |  |  |  |  |  |  |  |  |
|  | b |  |  |  |  |  |  |  |  |  |  |  |
|  | c |  |  |  |  |  |  |  |  |  |  |  |
|  | d |  |  |  |  |  |  |  |  |  |  |  |
| 7 | a | (6.7 years) | (6 years) | (7.6 years) | (12 years) | (7 years) | (35 years) | (10 years) | (16.3 years) | (22.6 years) | (12 years) |  |
|  | b |  |  |  |  |  |  |  |  |  |  | (2.3 years) |
| 8 | a |  |  |  |  |  |  |  |  |  |  |  |
|  | b |  |  |  |  |  |  |  |  | (90.1%) |  |  |
|  | c |  |  |  |  |  | (80.3%) |  |  |  |  |  |
|  | d |  |  |  |  |  |  |  |  |  |  |  |
| **Score** | | **5** | **7** | **7** | **8** | **8** | **8** | **6** | **7** | **7** | **8** | **7** |

**Table S4.** (cont.)

| **Section** | | **Hjorthoj 2015** | **Hoang 2011** | **Hoang 2013** | **Hoye 2016** | **Kay 1977** | **Kim 2018** | **Kodesh 2012** | **Laursen 2007** | **Laursen 2009** | **Laursen 2011** | **Laursen 2013** |
| --- | --- | --- | --- | --- | --- | --- | --- | --- | --- | --- | --- | --- |
| 1 | a | (Community, large sample) |  |  |  |  | (Community, large sample) | (Community, large sample) | (Community, large sample) | (Community, large sample) | (Community, large sample) | (Community, large sample) |
|  | b |  | (Nationwide) | (Nationwide) |  |  |  |  |  |  |  |  |
|  | c |  |  |  | (Small sample) | (Small sample) |  |  |  |  |  |  |
|  | d |  |  |  |  |  |  |  |  |  |  |  |
| 2 | a |  |  |  |  |  |  |  |  |  |  |  |
|  | b |  |  |  |  |  |  |  |  |  |  |  |
|  | c |  |  |  |  |  |  |  |  |  |  |  |
| 3 | a |  |  |  |  |  |  |  |  |  |  |  |
|  | b |  |  |  |  |  |  |  |  |  |  |  |
|  | c |  |  |  |  |  |  |  |  |  |  |  |
|  | d |  |  |  |  |  |  |  |  |  |  |  |
| 4 | a |  |  |  |  |  |  |  |  |  |  |  |
|  | b |  |  |  |  |  |  |  |  |  |  |  |
| 5 | a |  |  |  |  |  |  |  |  |  |  |  |
|  | b |  |  |  |  |  |  |  |  |  |  |  |
| 6 | a |  |  |  |  |  |  |  |  |  |  |  |
|  | b |  |  |  |  |  |  |  |  |  |  |  |
|  | c |  |  |  |  |  |  |  |  |  |  |  |
|  | d |  |  |  |  |  |  |  |  |  |  |  |
| 7 | a | (8.7 years) |  |  | (33 years) | (12 years) | (11 years) | (7 years) | (28 years) | (13 years) | (12 years) | (7 years) |
|  | b |  | (1 year) | (1 year) |  |  |  |  |  |  |  |  |
| 8 | a |  |  |  |  |  |  |  |  |  |  |  |
|  | b |  |  |  |  |  |  |  |  |  |  |  |
|  | c |  |  |  |  |  |  |  |  |  |  |  |
|  | d |  |  |  |  |  |  |  |  |  |  |  |
| **Score** | | **8** | **6** | **6** | **7** | **5** | **8** | **8** | **8** | **8** | **8** | **8** |

**Table S4.** (cont.)

| **Section** | | **Lomholt 2019** | **Medici 2015** | **Mohamed 2019** | **Newman 1991** | **Norton 1984** | **Osborn 2007/2008** | **Ösby 2001** | **Pan 2017** | **Pan 2020** | **Park 2019** | **Ramsey 2013** |
| --- | --- | --- | --- | --- | --- | --- | --- | --- | --- | --- | --- | --- |
| 1 | a | (Community, large sample) | (Community, large sample) | (Community, large sample) |  |  | (Community, large sample) |  | (Community, large sample) | (Community, large sample) | (Community, large sample) |  |
|  | b |  |  |  | (Community, small sample) |  |  | (Nationwide) |  |  |  | (Community, small sample) |
|  | c |  |  |  |  | (Lithium Users, community, small sample) |  |  |  |  |  |  |
|  | d |  |  |  |  |  |  |  |  |  |  |  |
| 2 | a |  |  |  |  |  |  |  |  |  |  |  |
|  | b |  |  |  |  |  |  |  |  |  |  |  |
|  | c |  |  |  |  |  |  |  |  |  |  |  |
| 3 | a |  |  |  |  |  |  |  |  |  |  |  |
|  | b |  |  |  |  |  |  |  |  |  |  |  |
|  | c |  |  |  |  |  |  |  |  |  |  |  |
|  | d |  |  |  |  |  |  |  |  |  |  |  |
| 4 | a |  |  |  |  |  |  |  |  |  |  |  |
|  | b |  |  |  |  |  |  |  |  |  |  |  |
| 5 | a |  |  |  |  |  |  |  |  |  |  |  |
|  | b |  |  |  |  |  |  |  |  |  |  |  |
| 6 | a |  |  |  |  |  |  |  |  |  |  |  |
|  | b |  |  |  |  |  |  |  |  |  |  |  |
|  | c |  |  |  |  |  |  |  |  |  |  |  |
|  | d |  |  |  |  |  |  |  |  |  |  |  |
| 7 | a | (20 years) | (17 years) | (10 years) | (10 years) | (10 years) | (15 years) | (23 years) |  |  | (11 years) | (18 years) |
|  | b |  |  |  |  |  |  |  | (3 years) | (3 years) |  |  |
| 8 | a |  |  |  |  |  |  |  |  |  |  |  |
|  | b |  |  |  |  |  |  |  |  |  |  |  |
|  | c |  |  |  |  |  |  |  |  |  |  |  |
|  | d |  |  |  |  |  |  |  |  |  |  |  |
| **Score** | | **8** | **8** | **8** | **7** | **8** | **8** | **7** | **6** | **7** | **7** | **6** |

# Table S4. (cont.)

| **Section** | | **Schaffer 2014** | **Schneider 2001** | **Schulman-Marcus 2016** | **Sharma 1994** | **Tsuang 1980** | **Vinogradova 2010** | **Webb 2014** | **Weeke 1987** | **Westman 2013** | **Yeh 2019** |
| --- | --- | --- | --- | --- | --- | --- | --- | --- | --- | --- | --- |
| 1 | a |  |  |  |  |  |  | (Community, large sample) |  |  |  |
|  | b | (Community, small sample) |  | (Large sample) |  |  |  |  |  | (Small sample) |  |
|  | c |  | (Small sample) |  | (Small sample) | (Small sample) | (Small sample, diabetes) |  | (Only male, small sample) |  | (Small sample) |
|  | d |  |  |  |  |  |  |  |  |  |  |
| 2 | a |  |  |  |  |  |  |  |  |  |  |
|  | b |  |  |  |  |  |  |  |  |  |  |
|  | c |  |  |  |  |  |  |  |  |  |  |
| 3 | a |  |  |  |  |  |  |  |  |  |  |
|  | b |  |  |  |  |  |  |  |  |  |  |
|  | c |  |  |  |  |  |  |  |  |  |  |
|  | d |  |  |  |  |  |  |  |  |  |  |
| 4 | a |  |  |  |  |  |  |  |  |  |  |
|  | b |  |  |  |  |  |  |  |  |  |  |
| 5 | a |  |  |  |  |  |  |  |  |  |  |
|  | b |  |  |  |  |  |  |  |  |  |  |
| 6 | a |  |  |  |  |  |  |  |  |  |  |
|  | b |  |  |  |  |  |  |  |  |  |  |
|  | c |  |  |  |  |  |  |  |  |  |  |
|  | d |  |  |  |  |  |  |  |  |  |  |
| 7 | a | (12 years) | (5 years) | (12 years) | (17 years) | (40 years) | (5 years) | 36 (years) |  | (20 years) | (13 years) |
|  | b |  |  |  |  |  |  |  | (4.5 years) |  |  |
| 8 | a |  |  |  |  |  |  |  |  |  |  |
|  | b |  | (99.4%) |  |  | (97%) |  |  |  |  |  |
|  | c |  |  |  | (86.4%) |  |  |  |  |  |  |
|  | d |  |  |  |  |  |  |  |  |  |  |
| **Score** | | **7** | **7** | **8** | **4** | **7** | **6** | **8** | **5** | **7** | 8 |

**Table S5.** GRADE evidence profile, summary of findings table.

| **Cause of mortality** | **Gender** | **№ of samples** | **Study design** | **Risk of bias** | **Inconsistency** | **Indirectness** | **Imprecision** | **Association leve** | **№ of Effect size Certainty Importance patients (95% CI)** |
| --- | --- | --- | --- | --- | --- | --- | --- | --- | --- |
|  | Total | 39 | Observational | Not seriousa,b,c | Not seriousd | Not serious | Not serious | Stronge,f,g | 450397h,i 2.02 ⨁⨁⨁! CRITICAL  (1.89 to 2.16) Moderate |
| **All causes** | Male | 23 | Observational | Not seriousa,b,c | Not seriousd | Not serious | Not serious | Strongg | 158752h,i 2.27 ⨁⨁⨁! CRITICAL  (2.13 to 2.43) Moderate |
|  | Female | 22 | Observational | Not seriousa,b,c | Not seriousd | Not serious | Not serious | Strongg | 215505h,i 2.26 ⨁⨁⨁! CRITICAL  (2.08 to 2.46) Moderate |
|  | Total | 16 | Observational | Not serious | Not seriousd | Not serious | Not serious | Noneg,j | 203181h,i 0.99 ⨁⨁!! IMPORTANT (0.88 to 1.11) Low |
| **Cancer** | Male | 9 | Observational | Not serious | Not seriousk | Not serious | Not serious | Noneg | 73004h,i 0.99 ⨁⨁!! IMPORTANT (0.93 to 1.06) Low |
|  | Female | 9 | Observational | Not serious | Not seriousd | Not serious | Not serious | Noneg | 104805h,i 1.03 ⨁⨁!! IMPORTANT (0.87 to 1.21) Low |
|  | Total | 27 | Observational | Not seriousa,c,l | Not seriousd | Not serious | Not serious | Noneg | 387963h,i 1.76 ⨁⨁!! IMPORTANT (1.53 to 2.02) Low |
| **Cardiovascular** | Male | 13 | Observational | Not seriousa,c,l | Not seriousd | Not serious | Not serious | Noneg | 96411h,i 1.82 ⨁⨁!! IMPORTANT (1.69 to 1.97) Low |
|  | Female | 14 | Observational | Not seriousa,c,l | Not seriousd | Not serious | Not serious | Noneg | 154499h,i 1.69 ⨁⨁!! IMPORTANT (1.44 to 1.99) Low |
|  | Total | 13 | Observational | Not serious | Not seriousd | Not serious | Not serious | Noneg | 165787h,i 1.57 ⨁⨁!! IMPORTANT (1.34 to 1.84) Low |
| **Cerebrovascular** | Male | 7 | Observational | Not serious | Not seriousk | Not serious | Not serious | Noneg | 56494h,i 1.70 ⨁⨁!! IMPORTANT (1.54 to 1.88) Low |
|  | Female | 7 | Observational | Not serious | Not seriousd | Not serious | Not serious | Noneg | 77620h,i 1.70 ⨁⨁!! IMPORTANT (1.30 to 2.23) Low |
|  | Total | 3 | Observational | Not serious | Not seriousd | Not serious | Seriousm | Strongg | 91341h,i 4.38 ⨁⨁!! IMPORTANT (1.51 to 12.70) Low |
| **Infectious** | Male | 2 | Observational | Not serious | Not seriousk | Not serious | Seriousm | Strongg | 6680h,i 3.24 ⨁⨁!! IMPORTANT (1.90 to 5.52) Low |
|  | Female | 2 | Observational | Not serious | Not seriousk | Not serious | Seriousm | Strongg,m | 8941h,i 2.62 ⨁⨁!! IMPORTANT (1.61 to 4.28) Low |

# l

**Table S6.** (cont.)

| **Cause of mortality** | **Gender** | **№ of samples** | **Study design** | **Risk of bias** | **Inconsistency** | **Indirectness** | **Imprecision** | **Association level** | **№ of Effect size Certainty Importance patients (95% CI)** |
| --- | --- | --- | --- | --- | --- | --- | --- | --- | --- |
|  | Total | 17 | Observational | Not serious | Not seriousd | Not serious | Not serious | Noneg | 354417h,i 1.91 ⨁⨁!! IMPORTANT (1.76 to 2.07) Low |
| Natural | Male | 11 | Observational | Not serious | Not seriousd | Not serious | Not serious | Strongg | 103783h,i 2.03 ⨁⨁⨁! IMPORTANT  (1.88 to 2.19) Moderate |
|  | Female | 12 | Observational | Not serious | Not seriousd | Not serious | Not serious | Strongg | 138050h,i 2.05 ⨁⨁⨁! IMPORTANT  (1.95 to 2.16) Moderate |
|  | Total | 6 | Observational | Not seriousl | Not seriousd | Not serious | Not serious | Strongg | 106661h,i 3.18 ⨁⨁⨁! IMPORTANT  (2.56 to 3.97) Moderate |
| Respiratory | Male | 5 | Observational | Not seriousl | Not seriousd | Not serious | Not serious | Strongg | 67192h,i 3.29 ⨁⨁⨁! IMPORTANT  (2.37 to 4.58) Moderate |
|  | Female | 5 | Observational | Not seriousl | Not seriousd | Not serious | Not serious | Strongg | 100247h,i 2.86 ⨁⨁⨁! IMPORTANT  (2.05 to 3.98) Moderate |
|  | Total | 25 | Observational | Not seriousa,c,l | Not seriousd | Not serious | Not serious | Very strongg | 180210h,i 11.69 ⨁⨁⨁⨁ CRITICAL (9.23 to 14.81) High |
| Suicide | Male | 11 | Observational | Not seriousa,c,l | Not seriousd | Not serious | Not serious | Very strongg | 47568h,i 14.02 ⨁⨁⨁⨁ CRITICAL (11.16 to 17.60) High |
|  | Female | 11 | Observational | Not seriousa,c,l | Not seriousd | Not serious | Not serious | Very strongg | 71175h,i 17.53 ⨁⨁⨁⨁ CRITICAL (10.76 to 28.54) High |
|  | Total | 17 | Observational | Not serious | Not seriousd | Not serious | Not serious | Very strongg | 349744h,i 7.29 ⨁⨁⨁⨁ CRITICAL  (6.42 to 8.28) High |
| Unnatural | Male | 10 | Observational | Not serious | Not seriousd | Not serious | Not serious | Very strongg | 103783h,i 6.69 ⨁⨁⨁⨁ CRITICAL  (5.85 to 7.66) High |
|  | Female | 11 | Observational | Not serious | Not seriousd | Not serious | Not serious | Very strongg | 138050h,i 9.33 ⨁⨁⨁⨁ CRITICAL (8.07 to 10.78) High |

a Included the study Ahrens 1995 - NOS (Newcastle-Otawa Scale) 5

b Included the study Kay 1977 - NOS (Newcastle-Otawa Scale) 5

c Included the study Weeke 1987 - NOS (Newcastle-Otawa Scale) 5

d Point estimates did not vary widely, confidence intervals substantially overlap, the direction of effect is consistent, but the magnitude of statistical heterogeneity (as measured by Q-test and I2 test) is high.

e Significant publication bias calculated by Egger`s test (0.036)

f Effect size > 2

g Not applicable

h Most of the effect sizes are standardized mortality ratios, which are calculated based on observed and expected deaths, not reported in some papers.

i Most of the effect sizes are standardized mortality ratios, which are calculated based on population databases and controls could not be estimated.

j Effect size < 2

k Point estimates did not vary widely, confidence intervals substantially overlap, the direction of effect is consistent, but the magnitude of statistical heterogeneity (as measured by Q-test and I2 test) is low.

l Included the study Sharma 1994: NOS (Newcastle-Otawa Scale) 4

m Large sample size, but small number of studies

**Table S7.** PRISMA 2020 Checklist

| **Section and Topic** | **Item #** | **Checklist item** | **Location where item is reported** |
| --- | --- | --- | --- |
| **TITLE** | | |  |
| Title | 1 | Identify the report as a systematic review. | p. 1 |
| **ABSTRACT** | | |  |
| Abstract | 2 | See the PRISMA 2020 for Abstracts checklist. | p.2 |
| **INTRODUCTION** | | |  |
| Rationale | 3 | Describe the rationale for the review in the context of existing knowledge. | p. 4 |
| Objectives | 4 | Provide an explicit statement of the objective(s) or question(s) the review addresses. | p. 5 |
| **METHODS** | | |  |
| Eligibility criteria | 5 | Specify the inclusion and exclusion criteria for the review and how studies were grouped for the syntheses. | p. 5 |
| Information sources | 6 | Specify all databases, registers, websites, organisations, reference lists and other sources searched or consulted to identify studies. Specify the date when each source was last searched or consulted. | p. 6 |
| Search strategy | 7 | Present the full search strategies for all databases, registers and websites, including any filters and limits used. | p. 6 and supp material |
| Selection process | 8 | Specify the methods used to decide whether a study met the inclusion criteria of the review, including how many reviewers screened each record and each report retrieved, whether they worked independently, and if applicable, details of automation tools used in the process. | p. 6 |
| Data collection process | 9 | Specify the methods used to collect data from reports, including how many reviewers collected data from each report, whether they worked independently, any processes for obtaining or confirming data from study investigators, and if applicable, details of automation tools used in the process. | p. 6 |
| Data items | 10a | List and define all outcomes for which data were sought. Specify whether all results that were compatible with each outcome domain in each study were sought (e.g., for all measures, time points, analyses), and if not, the methods used to decide which results to collect. | p. 7 |
|  | 10b | List and define all other variables for which data were sought (e.g. participant and intervention characteristics, funding sources). Describe any assumptions made about any missing or unclear information. | p. 7 |
| Study risk of bias assessment | 11 | Specify the methods used to assess risk of bias in the included studies, including details of the tool(s) used, how many reviewers assessed each study and whether they worked independently, and if applicable, details of automation tools used in the process. | p. 8 |
| Effect measures | 12 | Specify for each outcome the effect measure(s) (e.g. risk ratio, mean difference) used in the synthesis or presentation of results. | p. 6 |
| Synthesis methods | 13a | Describe the processes used to decide which studies were eligible for each synthesis (e.g. tabulating the study intervention characteristics and comparing against the planned groups for each synthesis (item #5)). | p. 7 |
|  | 13b | Describe any methods required to prepare the data for presentation or synthesis, such as handling of missing summary statistics, or data conversions. | p. 8 |
|  | 13c | Describe any methods used to tabulate or visually display results of individual studies and syntheses. | p. 7 |
|  | 13d | Describe any methods used to synthesize results and provide a rationale for the choice(s). If meta-analysis was performed, describe the model(s), method(s) to identify the presence and extent of statistical heterogeneity, and software package(s) used. | p. 7-8 |
|  | 13e | Describe any methods used to explore possible causes of heterogeneity among study results (e.g. subgroup analysis, meta-regression). | p. 8 |
|  | 13f | Describe any sensitivity analyses conducted to assess robustness of the synthesized results. | p. 7 |
| Reporting bias assessment | 14 | Describe any methods used to assess risk of bias due to missing results in a synthesis (arising from reporting biases). | p. 8 |
| Certainty assessment | 15 | Describe any methods used to assess certainty (or confidence) in the body of evidence for an outcome. | p. 8 |
| **RESULTS** | | |  |
| Study selection | 16a | Describe the results of the search and selection process, from the number of records identified in the search to the number of studies included in the review, ideally using a flow diagram. | pp. 9; 34 |
|  | 16b | Cite studies that might appear to meet the inclusion criteria, but which were excluded, and explain why they were excluded. | p. 34 |
| Study characteristics | 17 | Cite each included study and present its characteristics. | pp. 37-40 |
| Risk of bias in studies | 18 | Present assessments of risk of bias for each included study. | Supp. material |
| Results of individual studies | 19 | For all outcomes, present, for each study: (a) summary statistics for each group (where appropriate) and (b) an effect estimates and its precision (e.g. confidence/credible interval), ideally using structured tables or plots. | pp. 35-36; 41 |
| Results of syntheses | 20a | For each synthesis, briefly summarise the characteristics and risk of bias among contributing studies. | pp. 37-40 |
|  | 20b | Present results of all statistical syntheses conducted. If meta-analysis was done, present for each the summary estimate and its precision (e.g. confidence/credible interval) and measures of statistical heterogeneity. If comparing groups, describe the direction of the effect. | p. 12 |
|  | 20c | Present results of all investigations of possible causes of heterogeneity among study results. | p. 12 |
|  | 20d | Present results of all sensitivity analyses conducted to assess the robustness of the synthesized results. | p. 12 |
| Reporting biases | 21 | Present assessments of risk of bias due to missing results (arising from reporting biases) for each synthesis assessed. | p. 12 |
| Certainty of evidence | 22 | Present assessments of certainty (or confidence) in the body of evidence for each outcome assessed. | p. 12 |
| **DISCUSSION** | | |  |
| Discussion | 23a | Provide a general interpretation of the results in the context of other evidence. | p.13 |
|  | 23b | Discuss any limitations of the evidence included in the review. | p. 15 |
|  | 23c | Discuss any limitations of the review processes used. | p. 15 |
|  | 23d | Discuss implications of the results for practice, policy, and future research. | p. 17 |
| **OTHER INFORMATION** | | |  |
| Registration and protocol | 24a | Provide registration information for the review, including register name and registration number, or state that the review was not registered. | p. 5 |
|  | 24b | Indicate where the review protocol can be accessed, or state that a protocol was not prepared. | p. 5 |
|  | 24c | Describe and explain any amendments to information provided at registration or in the protocol. | NA |
| Support | 25 | Describe sources of financial or non-financial support for the review, and the role of the funders or sponsors in the review. | p.17 |
| Competing interests | 26 | Declare any competing interests of review authors. | p. 17 |
| Availability of data, code and other materials | 27 | Report which of the following are publicly available and where they can be found: template data collection forms; data extracted from included studies; data used for all analyses; analytic code; any other materials used in the review. | NA |

*From:*  Page MJ, McKenzie JE, Bossuyt PM, Boutron I, Hoffmann TC, Mulrow CD, et al. The PRISMA 2020 statement: an updated guideline for reporting systematic reviews. BMJ 2021;372:n71. doi: 10.1136/bmj.n71

For more information, visit: <http://www.prisma-statement.org/>

#
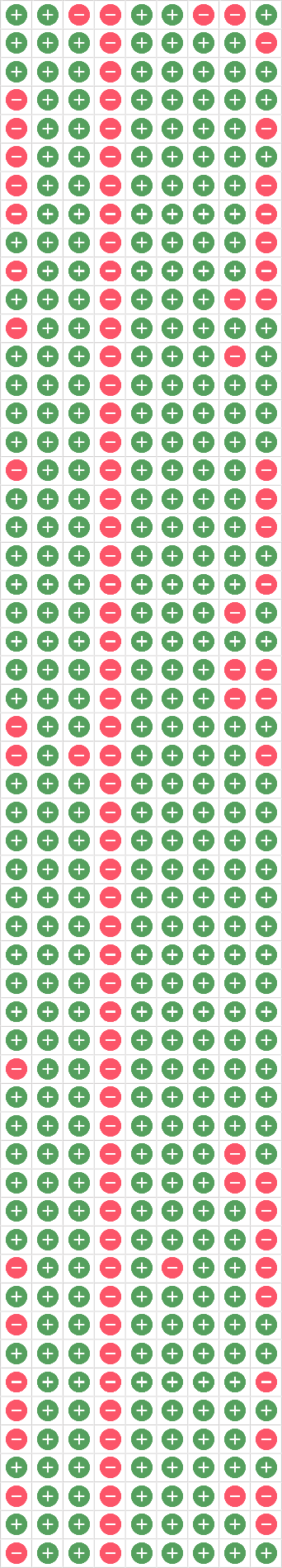
Nos question

**Study year** 1 2 3 4 5a 5b 6 7 8

Ahrens 1995

Ajetunmobi 2013

Almeida 2016

Angst 2002

Angst 2005

Angst 2013

Black 1987

Bratfos 1968

Callaghan 2014

Castagnini 2013

Chang 2010

Chang 2012

Chen 2010

Chen 2020

Choi 2019

Crump 2013

Dutta 2007

Fekadu 2015

Fiedorowicz 2009

Gale 2012

Guan 2013

Hayes 2017

Hjorthoj 2015

Hoang 2011

Hoang 2013

Hoye 2016

Kay 1977

Kim 2018

Kodesh 2012

Laursen 2007

Laursen 2009

Laursen 2011

Laursen 2013

Lomholt 2019

Medici 2015

Mohamed 2019

Newman 1991

Norton 1984 Osborn 2007/2008

Ösby 2001

Pan 2017

Pan 2020

Park 2019

Ramsey 2013

Saku 1995

Schaffer 2014

Schneider 2001

Schulman-Marcus 2016

Sharma 1994

Tsuang 1980

Vinogradova 2010

Webb 2014

Weeke 1987

Westman 2013

Yeh 2019

**Figure S1**. Risk of bias of individual studies.


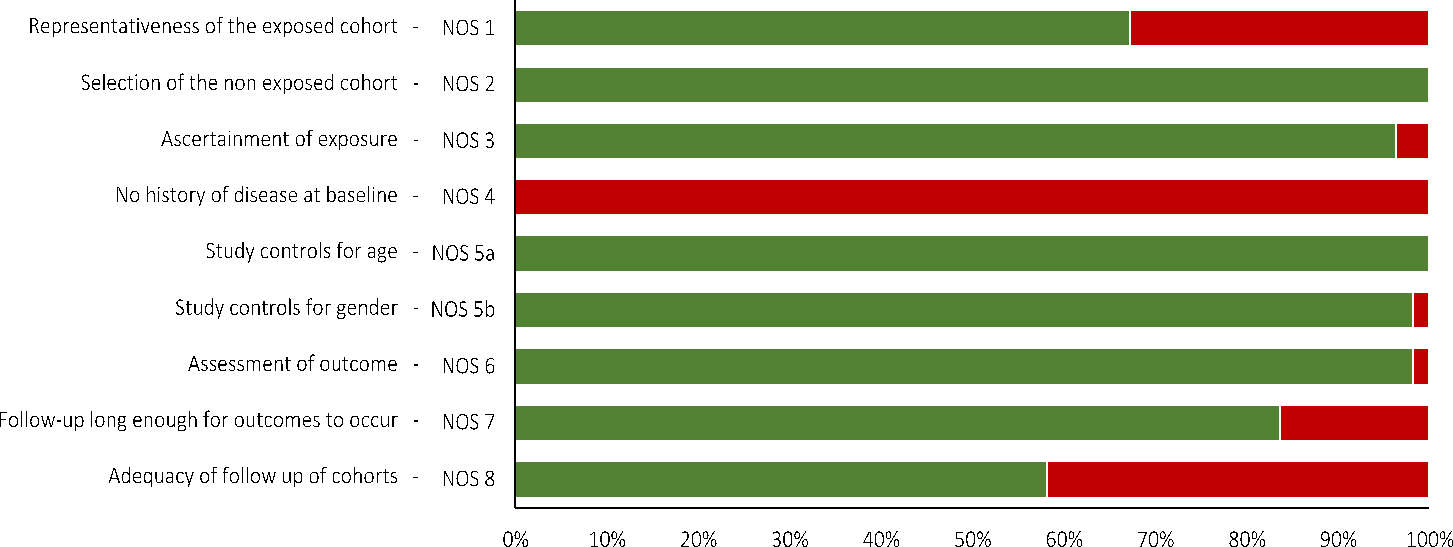


Figure S2. risk of bias of all included studies.

**Figure S3**. Prediction Intervals and its respective distribution*.

| **All-cause - Total** | **All-cause - Male** | **All-cause - Female** |
| --- | --- | --- |
| 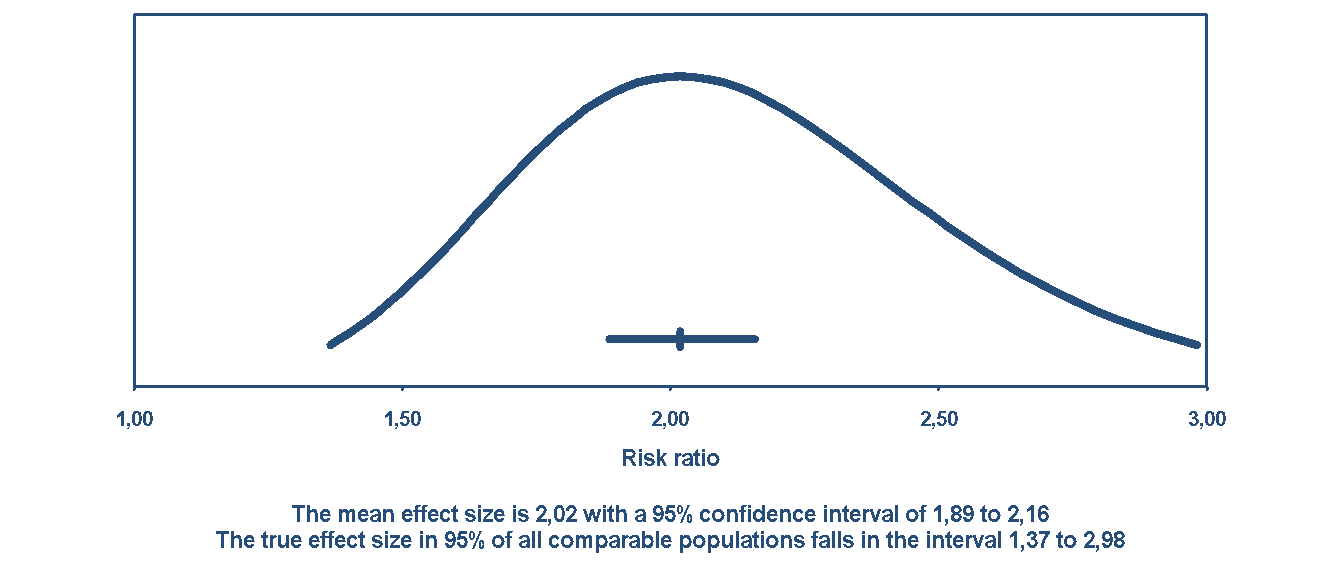 | 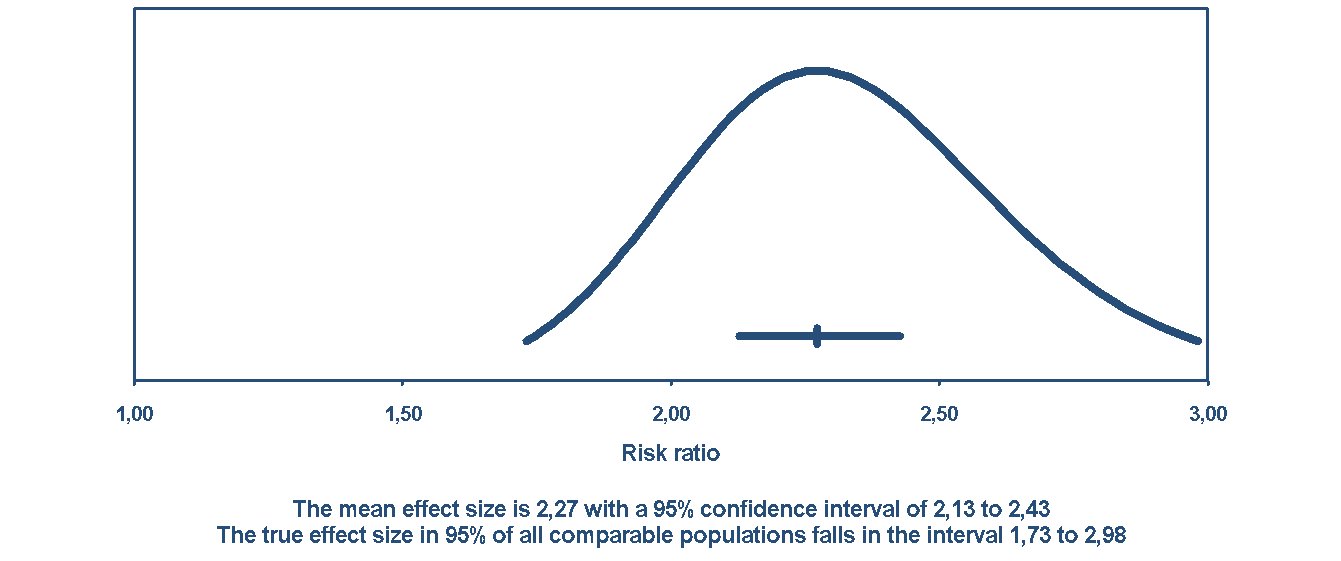 | 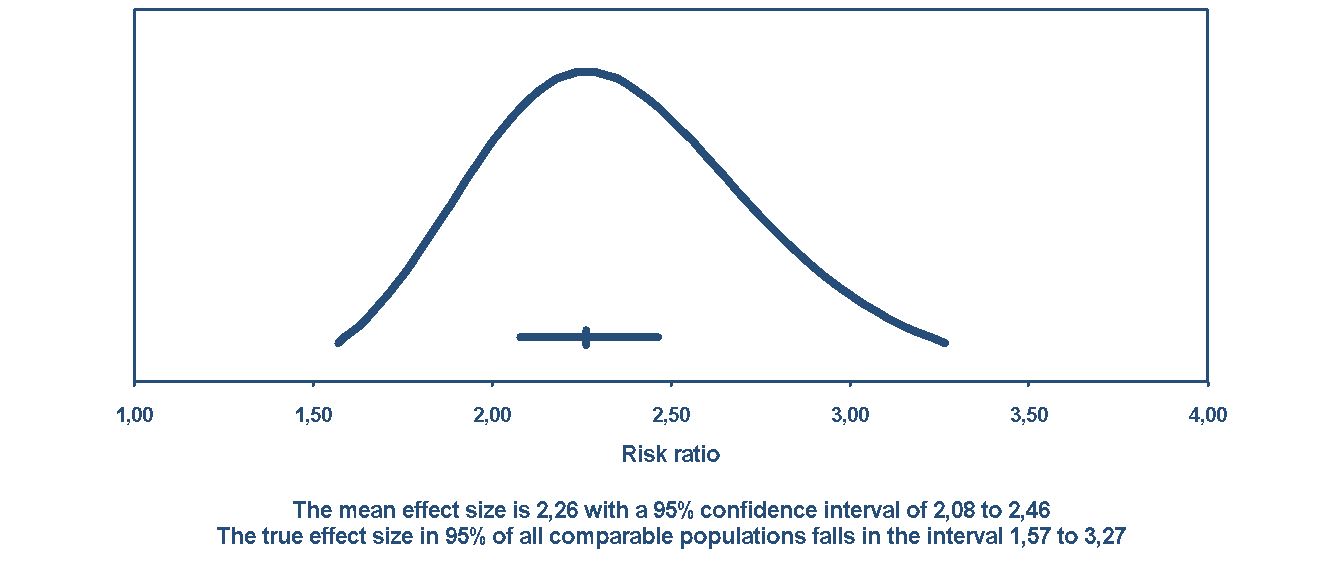 |
| **Cancer - Total** | **Cancer - Male** | **Cancer - Female** |
| 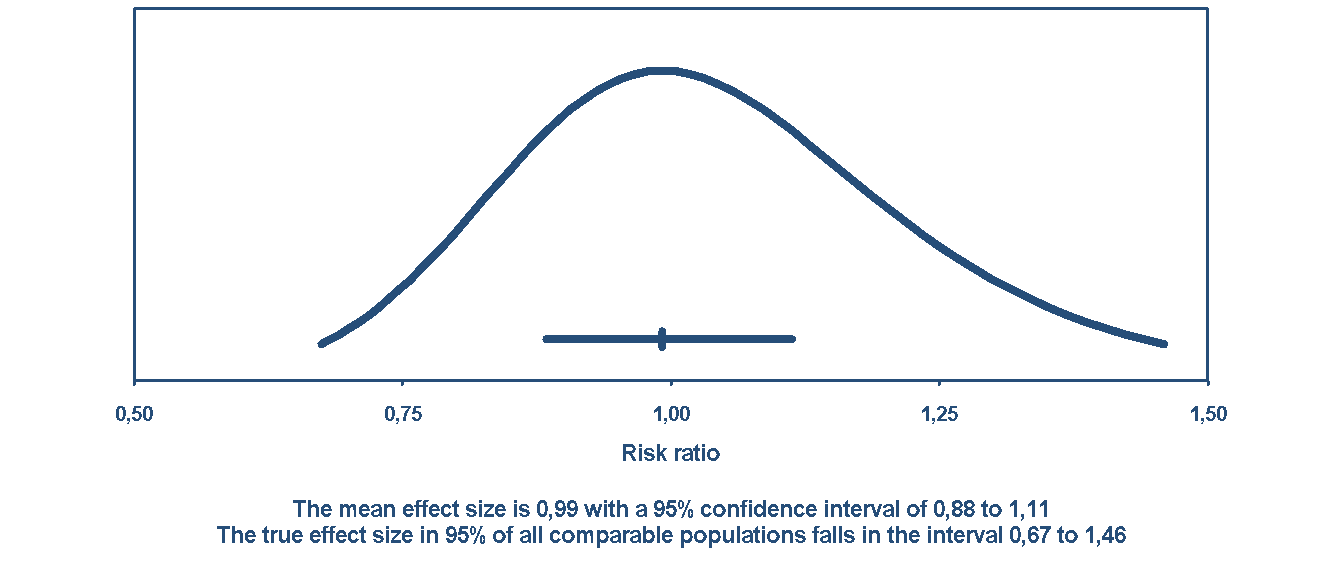 | 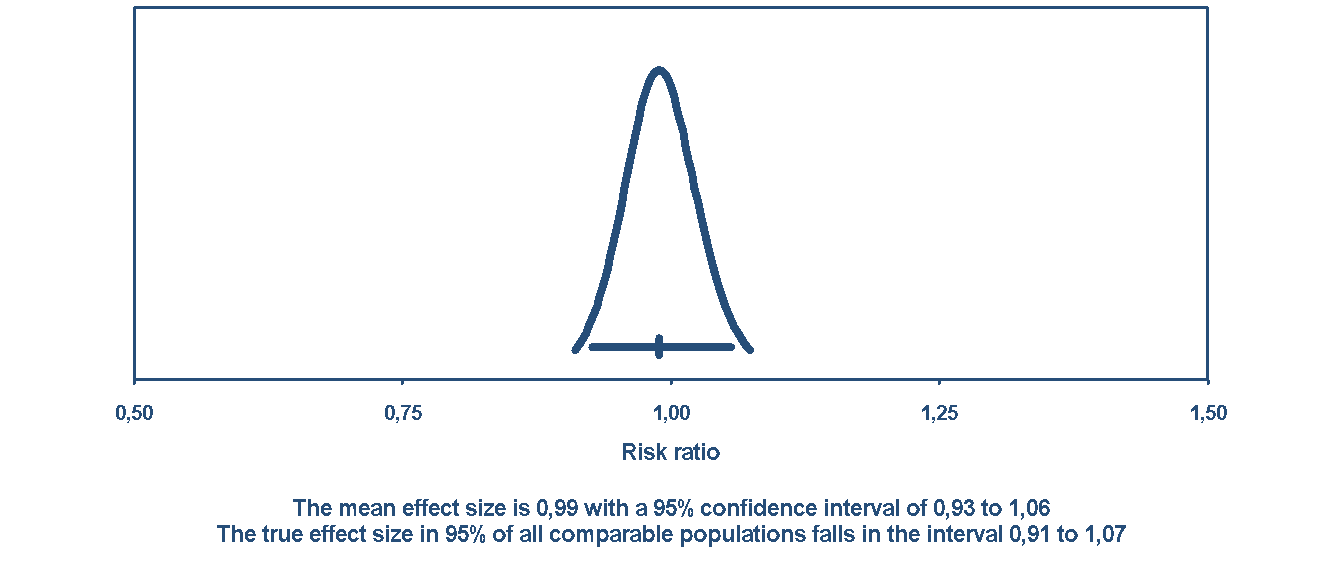 | 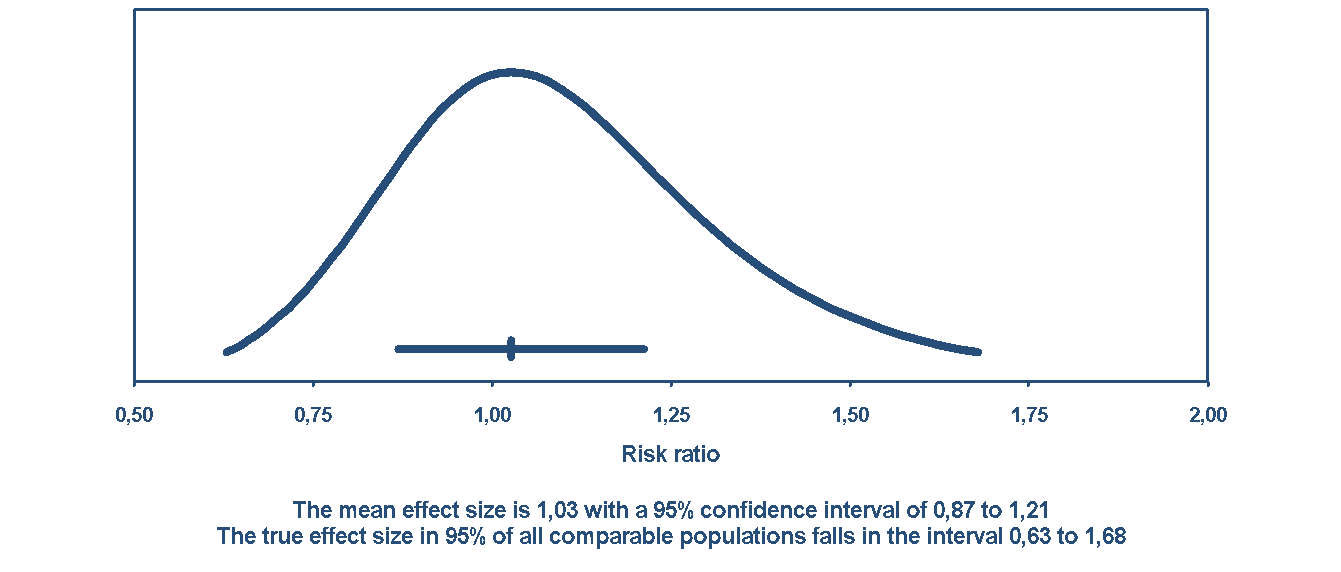 |
| **Cardiovascular - Total** | **Cardiovascular - Male** | **Cardiovascular - Female** |


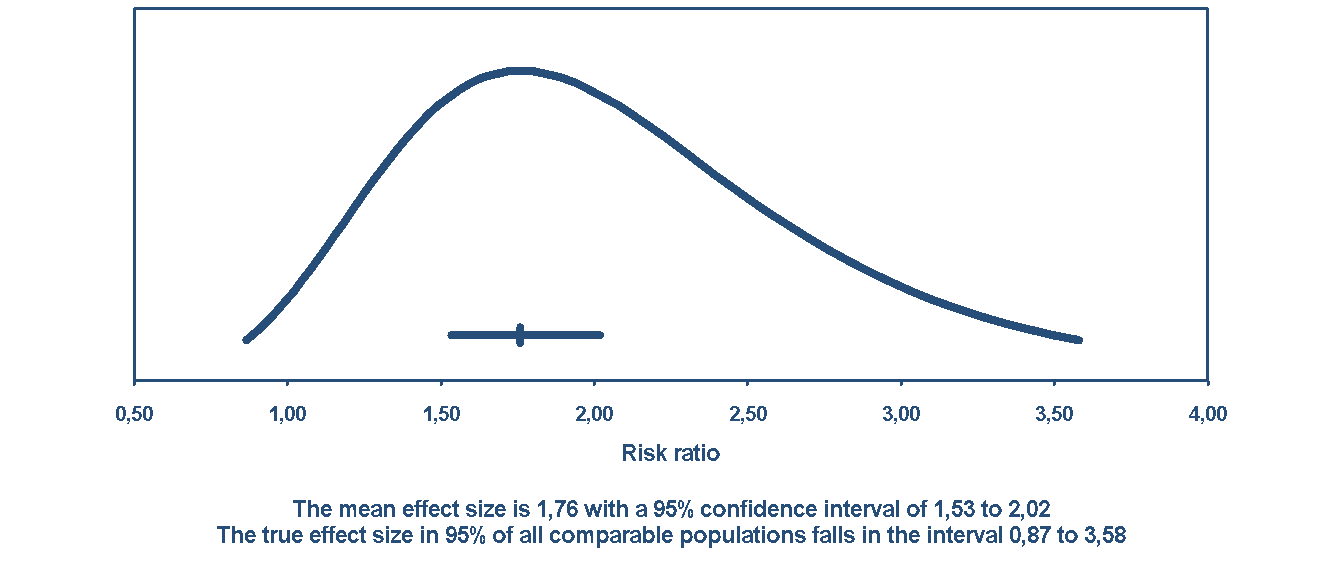

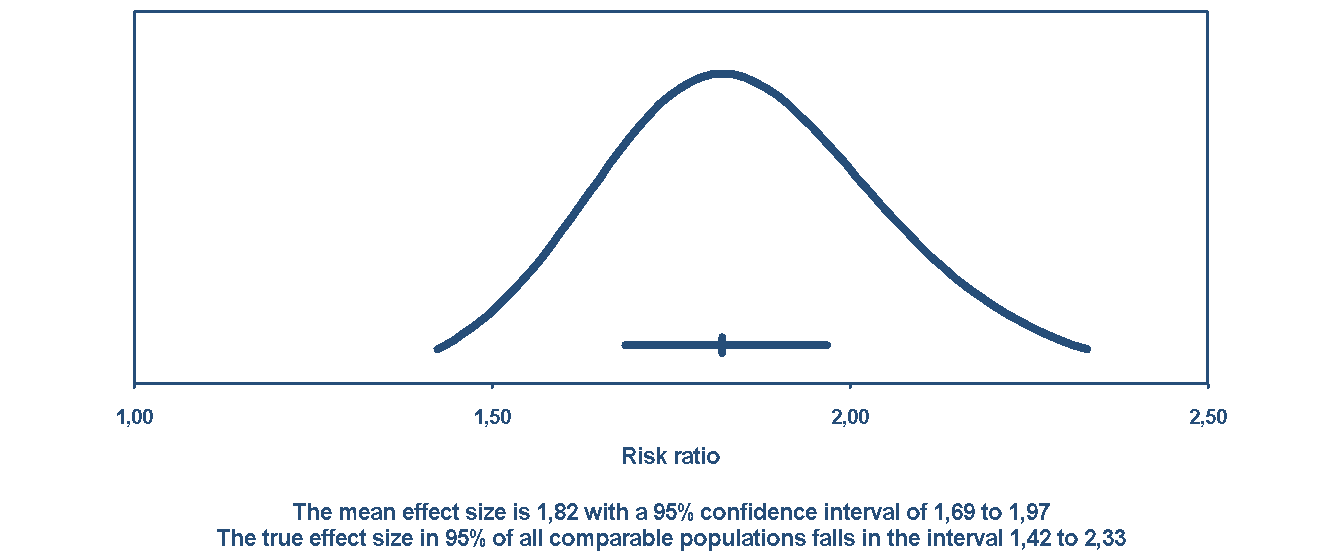

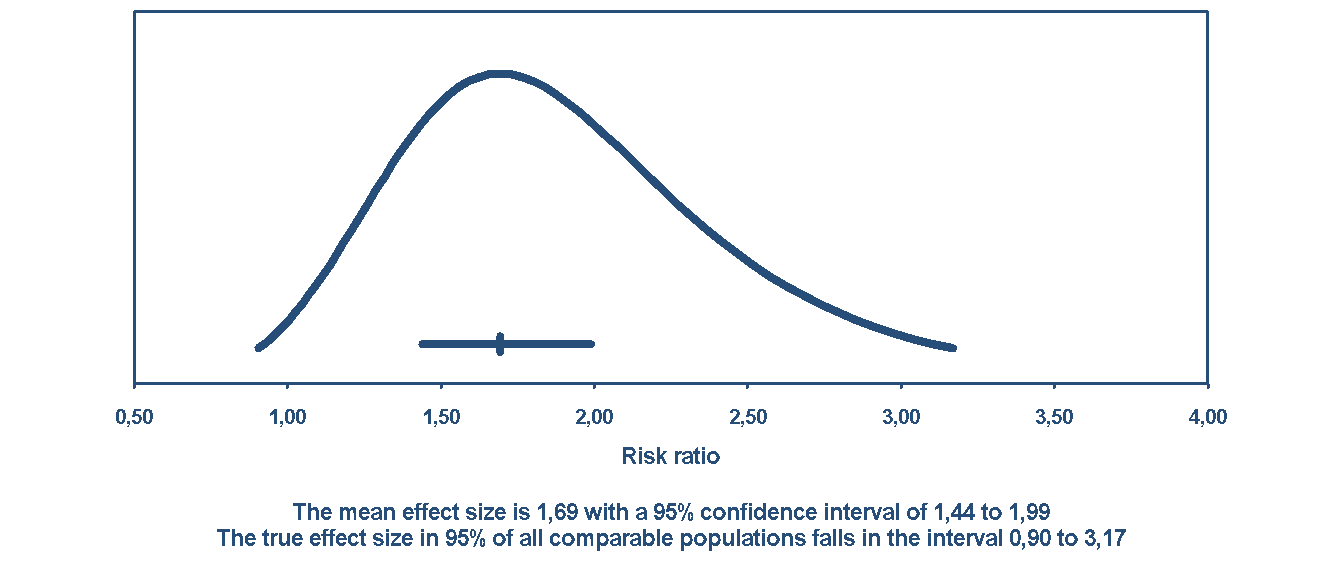


| **Figure S3.** (cont.). |  |  |
| --- | --- | --- |
| **Cerebrovascular - Total** | **Cerebrovascular - Male** | **Cerebrovascular - Female** |
| 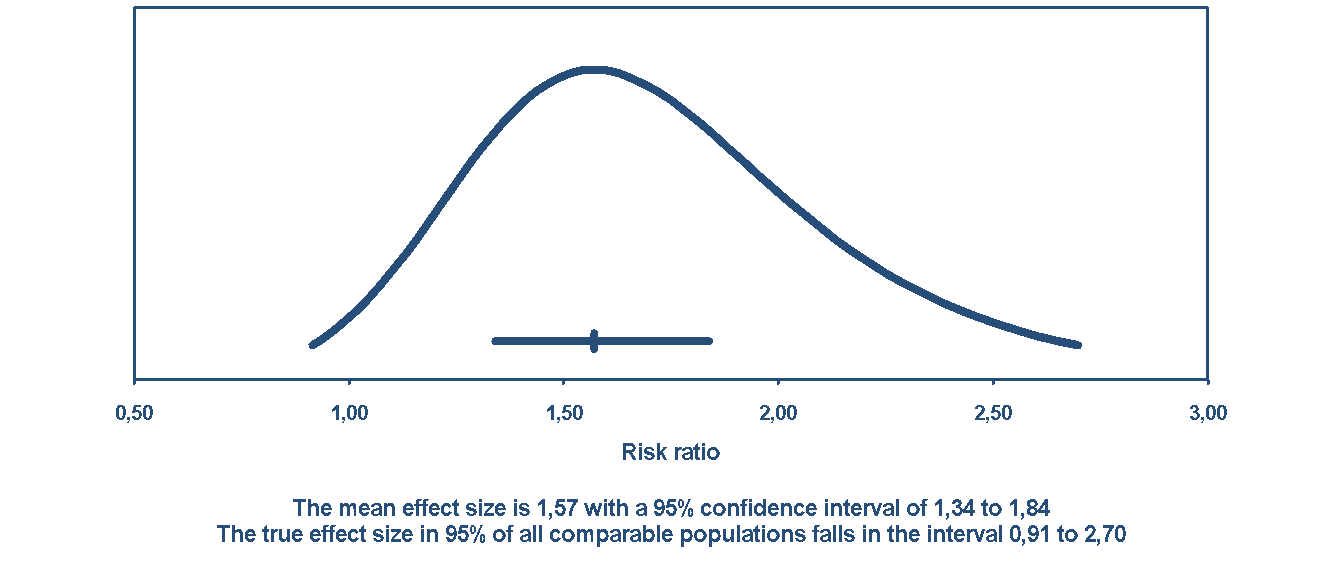 | 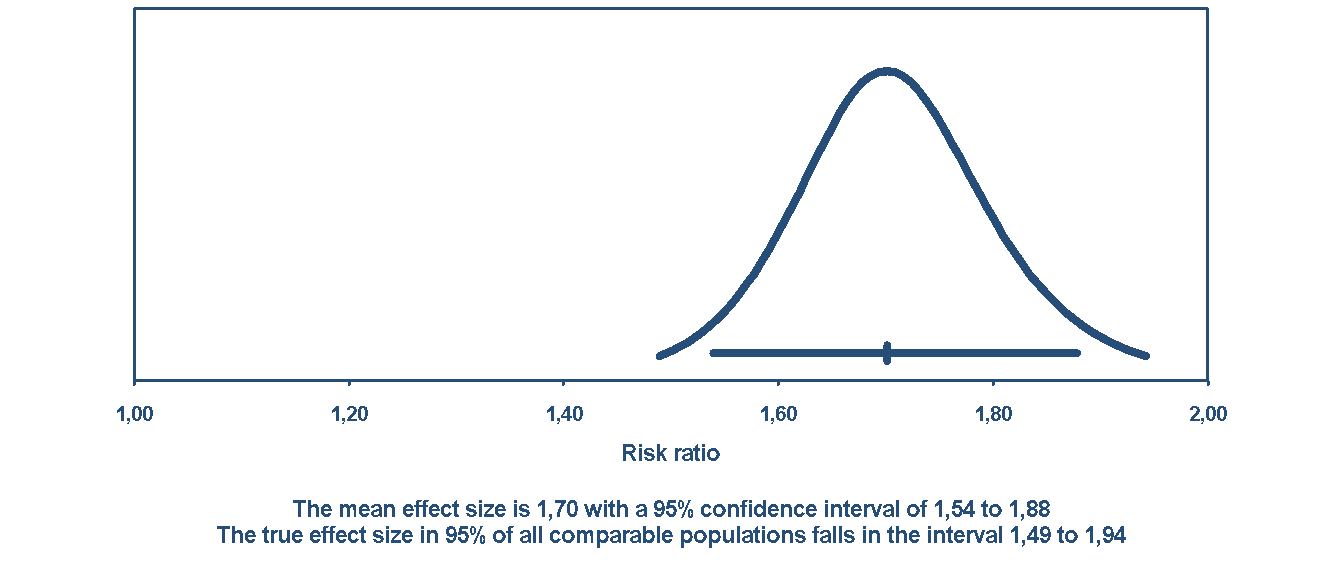 | 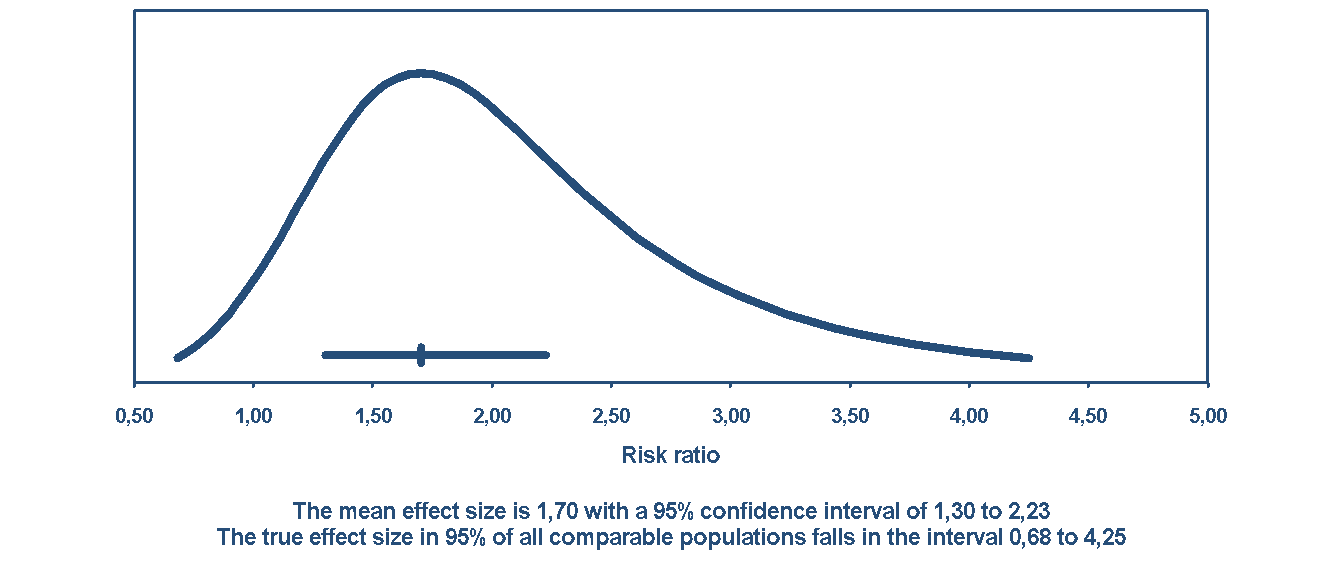 |
| **Natural - Total** | **Natural - Male** | **Natural - Female** |
| 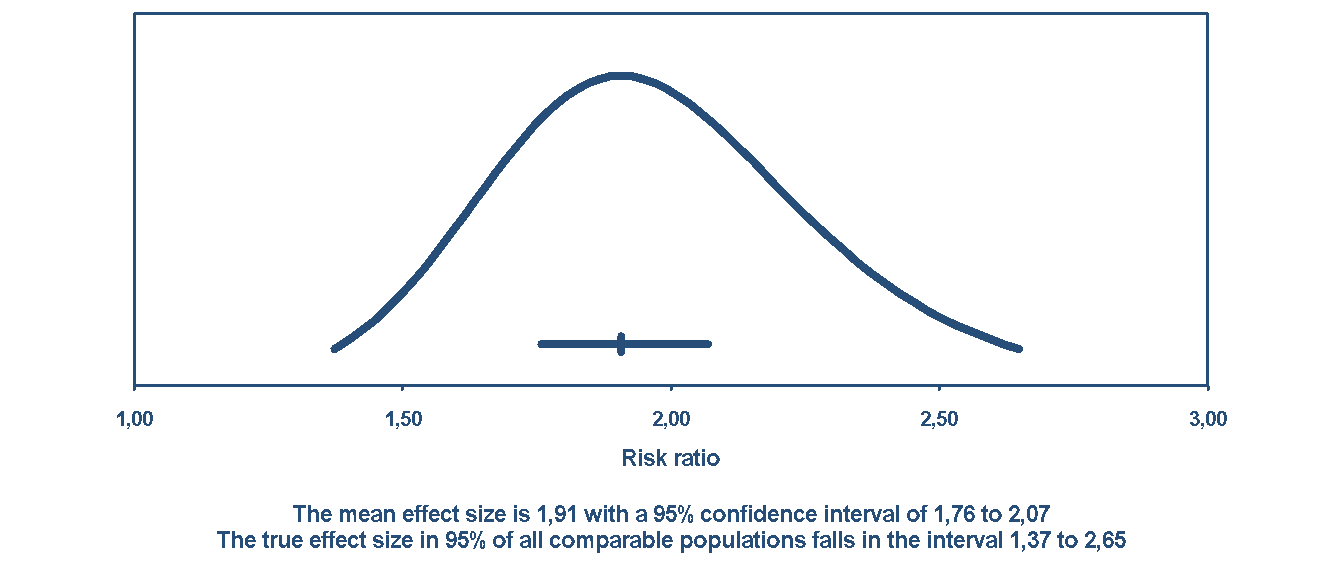 | 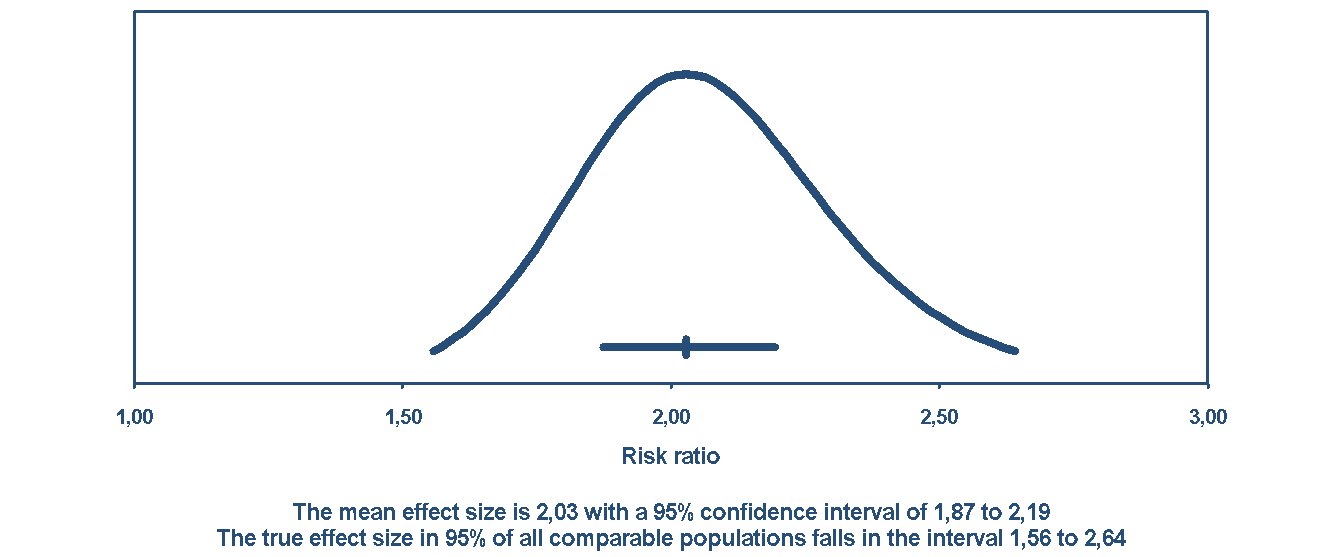 | 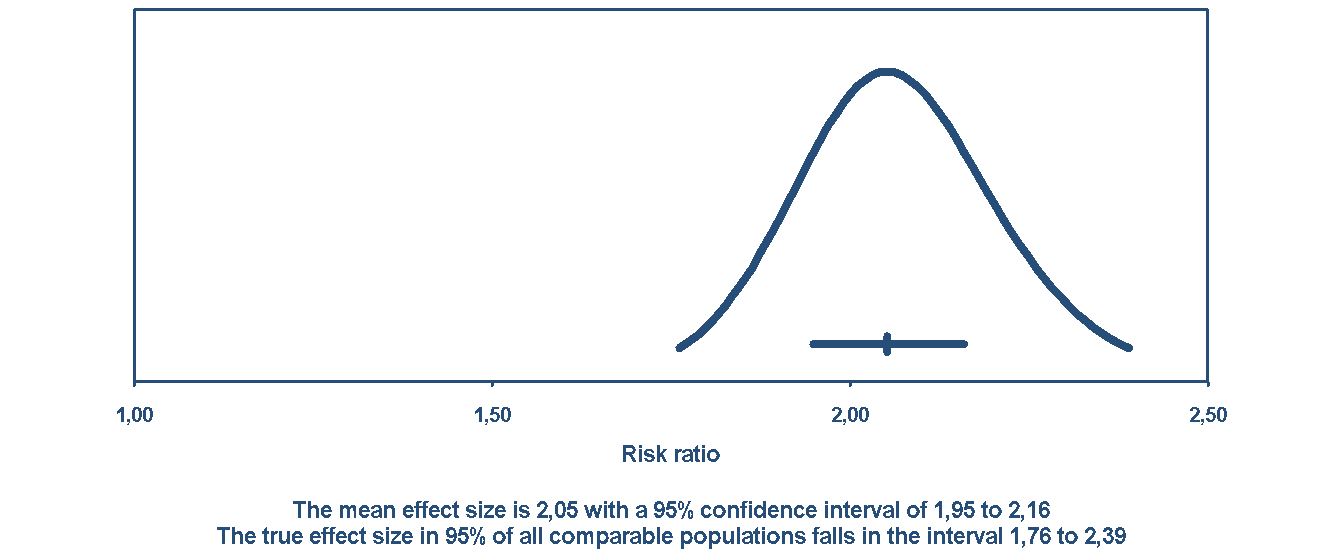 |
| **Respiratory - Total** | **Respiratory - Male** | **Respiratory - Female** |


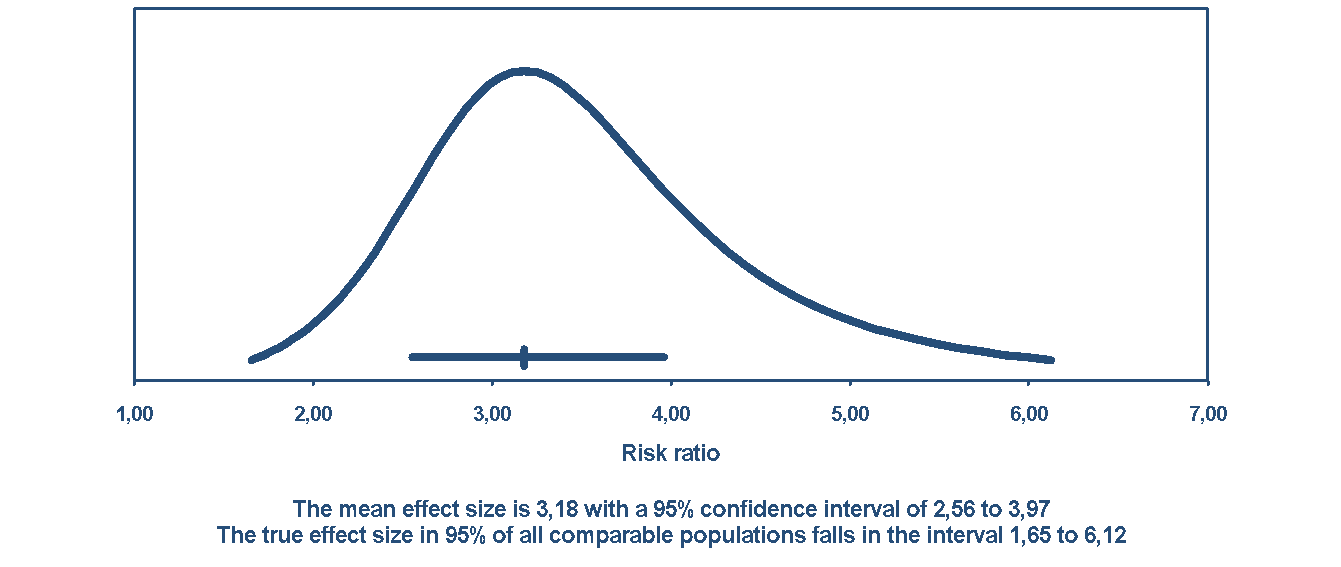

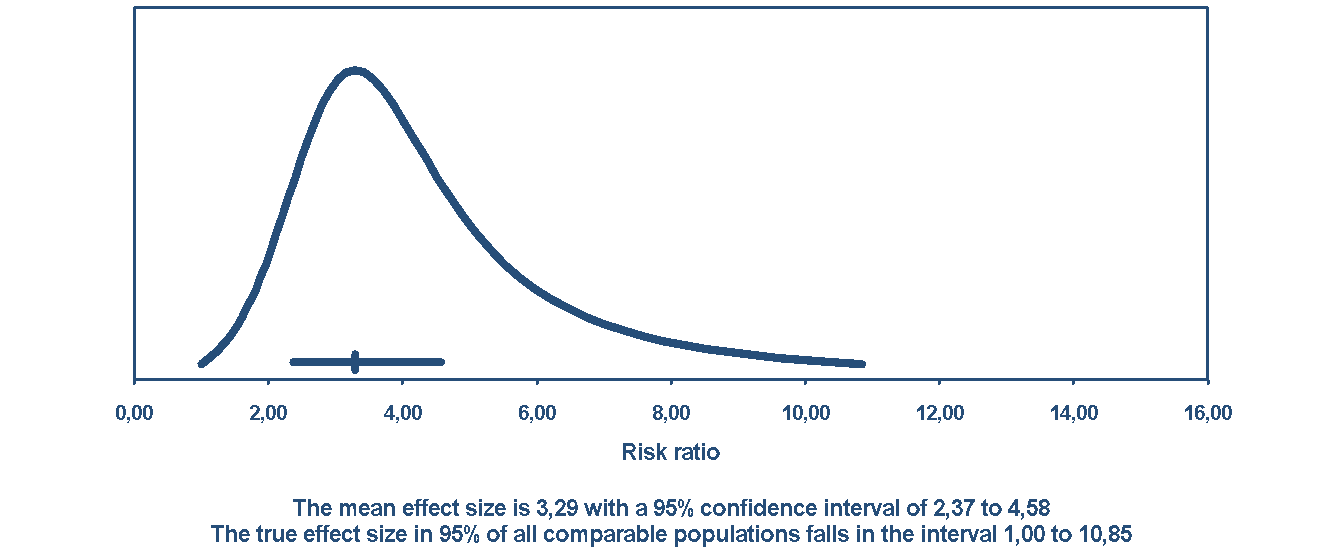

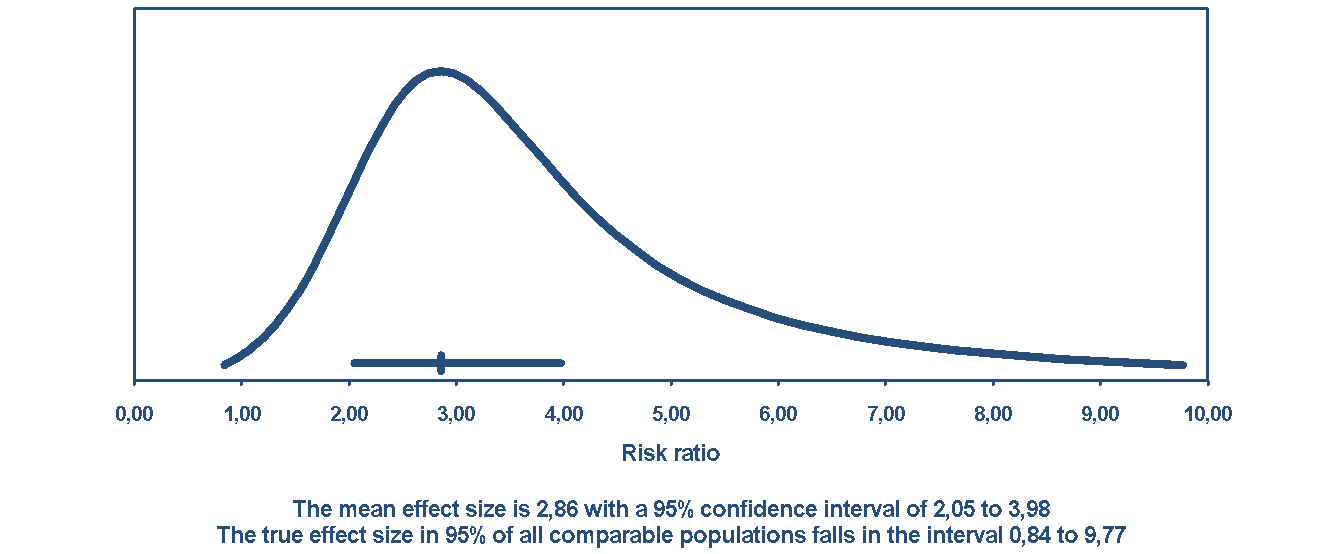


**Figure S3.** (cont.)


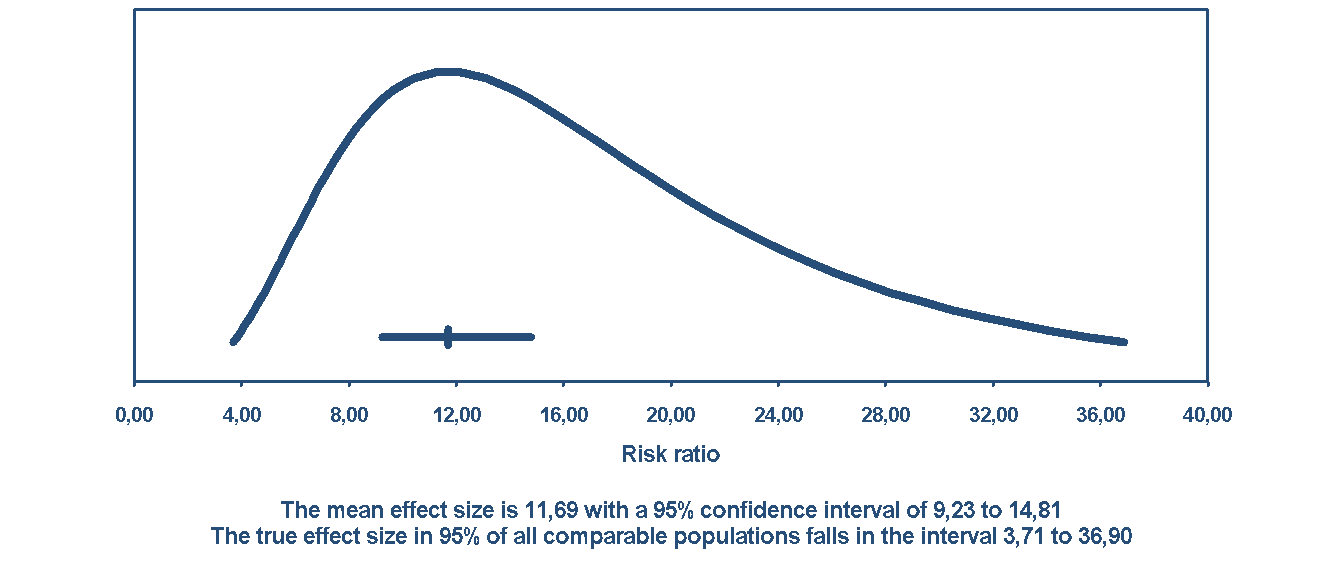

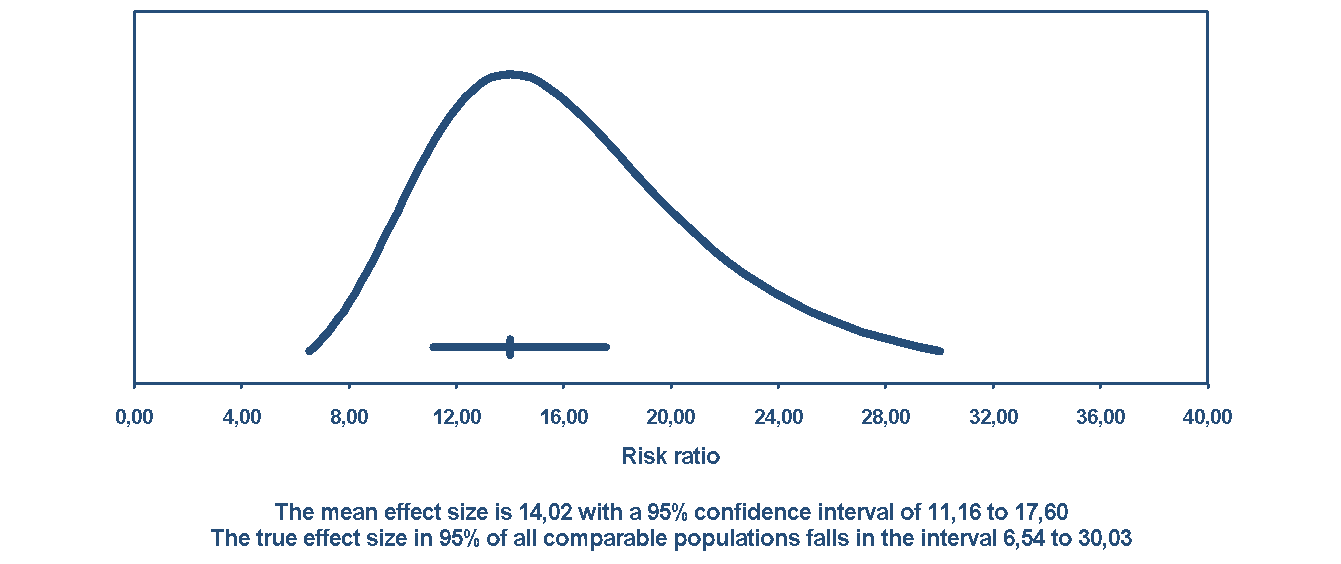

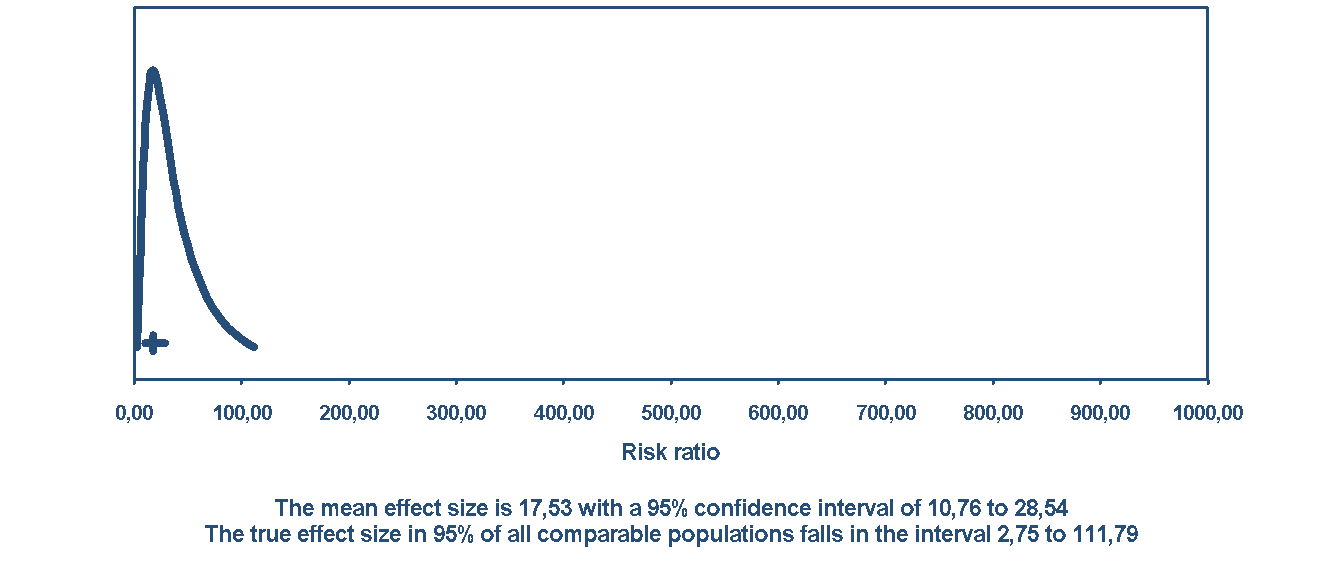

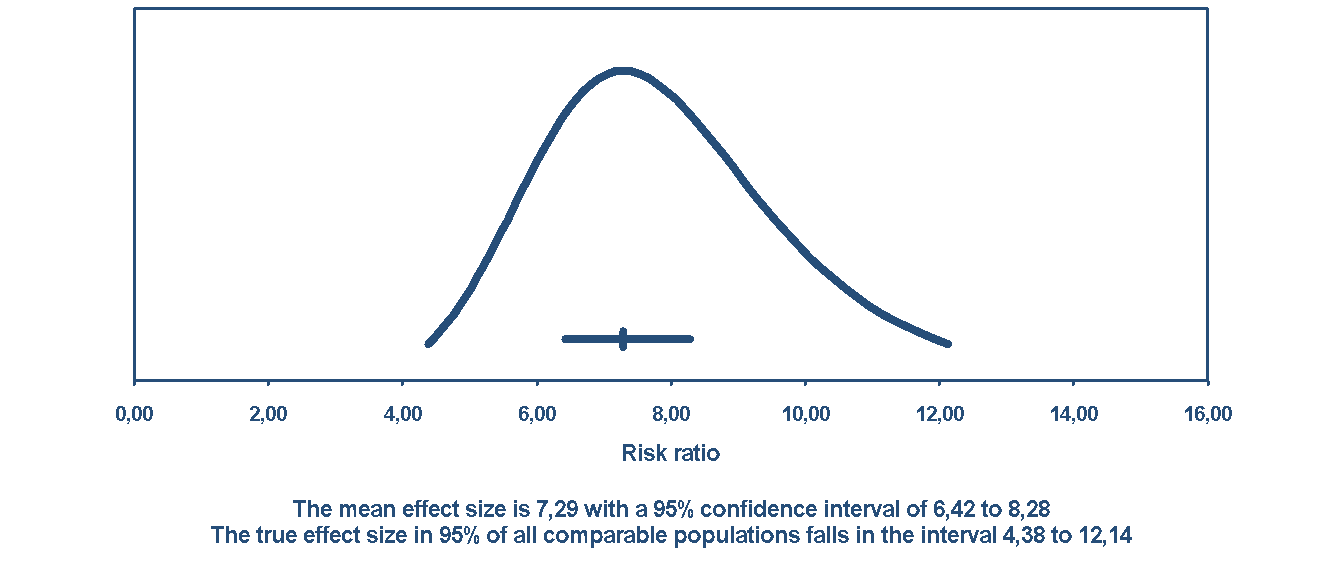

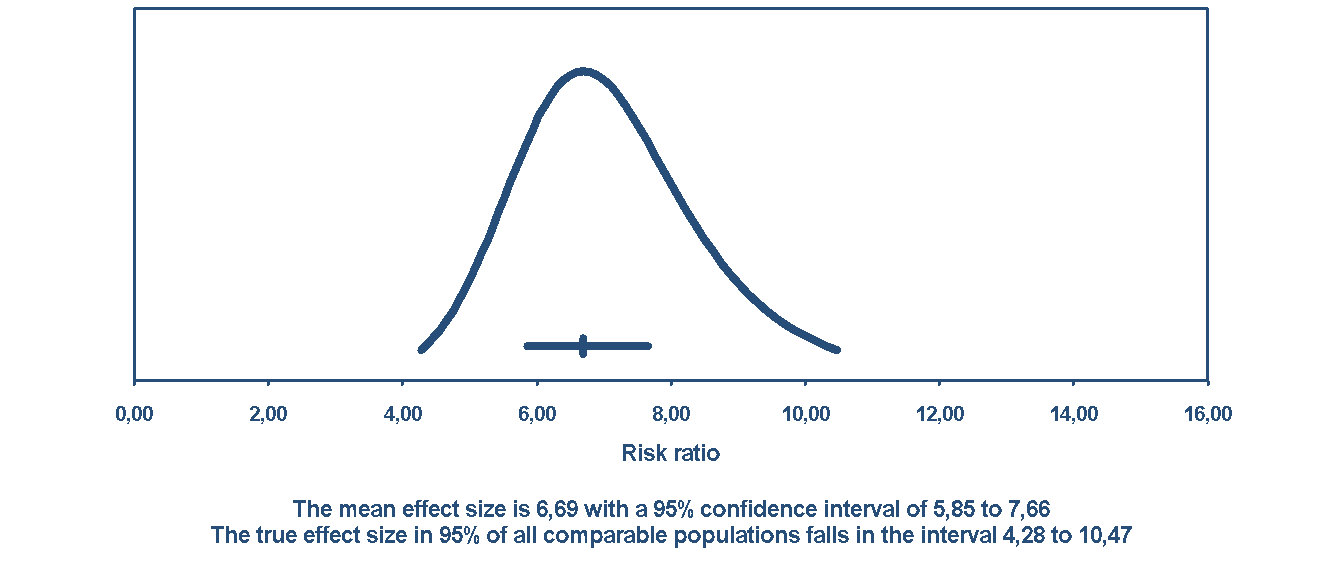

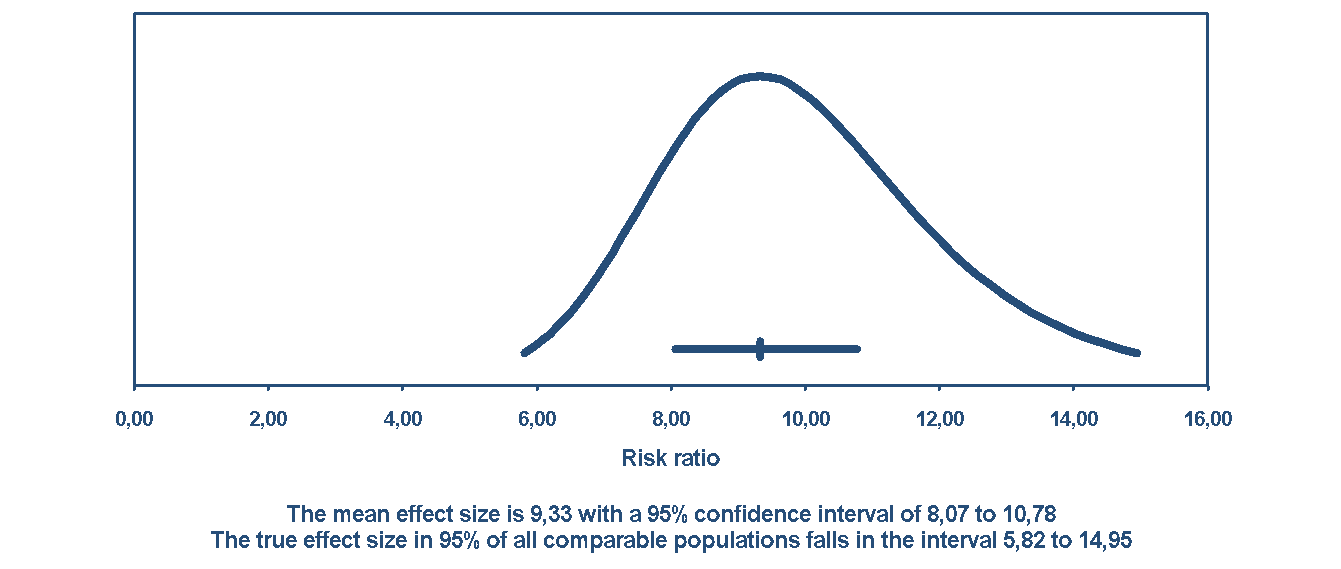


**Unnatural - Total Unnatural - Male Unnatural - Female**

**Suicide - Total Suicide - Male Suicide - Female**

*Extracted from the software Comprehensive Meta-analysis V3.

Standard Error

**Figure S4**. Funnel plot of all included studies in the analysis of overall mortality.


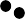

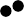

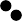

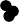


0

0.1

0.2

0.3

0.4

0.5

0.6

- 1.0

- 0.5

0

0.5

1.0

1.5

2

Log Effect Size

**References**

1. Ahrens B, Müller-Oerlinghausen B, Schou M, Wolf T. Excess cardiovascular and suicide mortality of affective disorders may be reduced by lithium prophylaxis. *J Affect Disord*. 1995;33(2):67-75. doi:<http://dx.doi.org/10.1016/0165-0327(94)00074-J>
2. Ajetunmobi O, Taylor M, Stockton D, Wood R. Early death in those previously hospitalised for mental healthcare in Scotland: a nationwide cohort study, 1986-2010. *BMJ Open*. 2013;3(7):9. doi:10.1136/bmjopen-2013-002768
3. Almeida OP, McCaul K, Hankey GJ, Yeap BB, Golledge J, Flicker L. Suicide in older men: The health in men cohort study (HIMS). *Prev Med (Baltim)*. 2016;93((Almeida O.P., osvaldo.almeida@uwa.edu.au) School of Psychiatry & Clinical Neurosciences, University of Western Australia, Perth, Australia(Almeida O.P., osvaldo.almeida@uwa.edu.au; McCaul K.; Flicker L.) WA Centre for Health & Ageing of Centre for Medica):33-38. doi:10.1016/j.ypmed.2016.09.022
4. Angst F, Stassen HH, Clayton PJ, Angst J. Mortality of patients with mood disorders: follow-up over 34-38 years. *J Affect Disord*. 2002;68(2-3):167-181. [http://ezproxy.libproxy.db.erau.edu/login?url=https://www.proquest.com/docview/71810101?acco](http://ezproxy.libproxy.db.erau.edu/login?url=https%3A//www.proquest.com/docview/71810101%3Facco) untid=27203
5. Angst J, Angst F, Gerber-Werder R, Gamma A. Suicide in 406 mood-disorder patients with and without long-term medication: A 40 to 44 years’ follow-up. *Archives of Suicide Research*. 2005;9(3):279-300. doi:10.1080/13811110590929488
6. Angst J, Hengartner MP, Gamma A, D VZ, Angst F. Mortality of 403 patients with mood disorders 48 to 52 years after their psychiatric hospitalisation. *Eur Arch Psychiatry Clin Neurosci*. 2013;263(5):425-

434. doi:10.1007/s00406-012-0380-1

1. Black DW, Winokur G, Nasrallah A. Mortality in patients with primary unipolar depression, secondary unipolar depression, and bipolar affective disorder: A comparison with general population mortality. *Int J Psychiatry Med*. 1987;17(4):351-360. doi:<http://dx.doi.org/10.2190/VL1B-> 7YEE-91J5-MWRA
2. Bratfos O, Haug JO. The course of manic-depressive psychosis: A follow-up investigation of 215 patients. *Acta Psychiatr Scand*. 1968;44(1):89-112. doi:<http://dx.doi.org/10.1111/j.1600-> 0447.1968.tb07637.x
3. Callaghan RC, Veldhuizen S, Jeysingh T, et al. Patterns of tobacco-related mortality among individuals diagnosed with schizophrenia, bipolar disorder, or depression. *J Psychiatr Res*. 2014;48(1):102-110. doi:10.1016/j.jpsychires.2013.09.014
4. Castagnini A, Foldager L, Bertelsen A. Excess mortality of acute and transient psychotic disorders: Comparison with bipolar affective disorder and schizophrenia. *Acta Psychiatr Scand*. 2013;128(5):370-375. doi:10.1111/acps.12077
5. C.-K. C, Hayes RD, Broadbent M, et al. All-cause mortality among people with serious mental illness (SMI), substance use disorders, and depressive disorders in southeast London: A cohort study. *BMC Psychiatry*. 2010;10((Chang C.-K., chin-kuo.chang@kcl.ac.uk; Hayes R.D., richard.hayes@kcl.ac.uk; Broadbent M., matthew.broadbent@kcl.ac.uk; Fernandes A.C., andrea.1.fernandes@kcl.ac.uk; Stewart R., robert.stewart@kcl.ac.uk) King’s College London, Section of Epidemiology, Dep). doi:10.1186/1471-244X-10-77
6. J.-C. C, H.-H. C, Yen AMF, Chen SLS, C.-S. L. Survival of bipolar depression, other type of depression and comorbid ailments: Ten-year longitudinal follow-up of 10,922 Taiwanese patients with depressive disorders (KCIS no. PSY1). *J Psychiatr Res*. 2012;46(11):1442-1448. doi:10.1016/j.jpsychires.2012.07.014
7. Chen YH, Lee HC, Lin HC. Mortality among psychiatric patients in Taiwan-Results from a universal National Health Insurance programme. *Psychiatry Res*. 2010;178(1):160-165. doi:10.1016/j.psychres.2008.07.023
8. P.-H. C, S.-Y. T, C.-H. P, et al. Incidence and risk factors of sudden cardiac death in bipolar disorder across the lifespan. *J Affect Disord*. 2020;274((Chen P.-H.; Tsai S.-Y.) Department of Psychiatry, Taipei Medical University Hospital, Taipei, Taiwan(Chen P.-H.; Tsai S.-Y.; Kuo C.-J., tcpckuo@seed.net.tw) Psychiatric Research Center, Taipei Medical University Hospital, Taipei, Taiwan(Chen P.-H.; Tsai):210-

217. doi:10.1016/j.jad.2020.05.094

1. Choi JW, Lee KS, Kim TH, Choi J, Han E. Suicide risk after discharge from psychiatric care in South Korea. *J Affect Disord*. 2019;251:287-292. doi:10.1016/j.jad.2019.03.079
2. Crump C, Sundquist K, Winkleby MA, Sundquist J. Comorbidities and mortality in bipolar disorder: A Swedish national cohort study. *JAMA Psychiatry*. 2013;70(9):931-939. doi:10.1001/jamapsychiatry.2013.1394
3. Dutta R, Boydell J, Kennedy N, J VO, Fearon P, Murray RM. Suicide and other causes of mortality in bipolar disorder: A longitudinal study. *Psychol Med*. 2007;37(6):839-847. doi:10.1017/S0033291707000347
4. Fekadu A, Medhin G, Kebede D, et al. Excess mortality in severe mental illness: 10-year population- based cohort study in rural Ethiopia. *The British Journal of Psychiatry*. 2015;206(4):289-296. doi:<http://dx.doi.org/10.1192/bjp.bp.114.149112>
5. Fiedorowicz JG, Solomon DA, Endicott J, et al. Manic/hypomanic symptom burden and cardiovascular mortality in bipolar disorder. *Psychosom Med*. 2009;71(6):598-606. doi:10.1097/PSY.0b013e3181acee26
6. Gale CR, Batty GD, Osborn DPJ, Tynelius P, Whitley E, Rasmussen F. Association of mental disorders in early adulthood and later psychiatric hospital admissions and mortality in a cohort study of more than 1 million men. *Arch Gen Psychiatry*. 2012;69(8):823-831. doi:10.1001/archgenpsychiatry.2011.2000
7. Guan NC, Termorshuizen F, Laan W, et al. Cancer mortality in patients with psychiatric diagnoses: A higher hazard of cancer death does not lead to a higher cumulative risk of dying from cancer. *Social Psychiatry and Psychiatric Epidemiology: The International Journal for Research in Social and Genetic Epidemiology and Mental Health Services*. 2013;48(8):1289-1295. doi:<http://dx.doi.org/10.1007/s00127-012-0612-8>
8. Hayes JF, Marston L, Walters K, King MB, Osborn DPJ. Mortality gap for people with bipolar disorder and schizophrenia: UK-based cohort study 2000-2014. *British Journal of Psychiatry*. 2017;211(3):175-

181. doi:10.1192/bjp.bp.117.202606

1. Hjorthøj C, Østergaard MLD, Benros ME, et al. Association between alcohol and substance use disorders and all-cause and cause-specific mortality in schizophrenia, bipolar disorder, and unipolar depression: A nationwide, prospective, register-based study. *Lancet Psychiatry*. 2015;2(9):801-808. doi:10.1016/S2215-0366(15)00207-2
2. Hoang U, Stewart R, Goldacre MJ. Mortality after hospital discharge for people with schizophrenia or bipolar disorder: retrospective study of linked English hospital episode statistics, 1999-2006. *BMJ : British Medical Journal (Online)*. 2011;343. doi:<http://dx.doi.org/10.1136/bmj.d5422>
3. Hoang U, Goldacre MJ, Stewart R. Avoidable mortality in people with schizophrenia or bipolar disorder in England. *Acta Psychiatr Scand*. 2013;127(3):195-201. doi:10.1111/ACPS.12045
4. Høye A, Nesvåg R, Reichborn-Kjennerud T, Jacobsen BK. Sex differences in mortality among patients admitted with affective disorders in North Norway: A 33-year prospective register study. *Bipolar Disord*. 2016;18(3):272-281. doi:10.1111/bdi.12389
5. Kay DWK, Petterson U. VI. MORTALITY. *Acta Psychiatr Scand*. 1977;56(S269):55-60. doi:10.1111/j.1600-0447.1977.tb10824.x
6. Kim W, S.-Y. J, T.-H. L, Lee JE, E.-C. P. Association between continuity of care and subsequent hospitalization and mortality in patients with mood disorders: Results from the Korea National Health Insurance cohort. *PLoS One*. 2018;13(11). doi:10.1371/journal.pone.0207740
7. Kodesh A, Goldshtein I, Gelkopf M, Goren I, Chodick G, Shalev V. Epidemiology and comorbidity of severe mental illnesses in the community: findings from a computerized mental health registry in a large Israeli health organization. *Soc Psychiatry Psychiatr Epidemiol*. 2012;47(11):1775-1782. <http://www.embase.com/search/results?subaction=viewrecord&from=export&id=L366376947>
8. Laursen TM, Munk-Olsen T, Nordentoft M, Mortensen PB. Increased mortality among patients admitted with major psychiatric disorders: A register-based study comparing mortality in unipolar depressive disorder, bipolar affective disorder, schizoaffective disorder, and schizophrenia. *J Clin Psychiatry*. 2007;68(6):899-907. doi:10.4088/JCP.v68n0612
9. Laursen TM, Munk-Olsen T, Agerbo E, Gasse C, Mortensen PB. Somatic hospital contacts, invasive cardiac procedures, and mortality from heart disease in patients with severe mental disorder. *Arch Gen Psychiatry*. 2009;66(7):713-720. doi:10.1001/archgenpsychiatry.2009.61
10. Laursen TM, Munk-Olsen T, Gasse C. Chronic Somatic Comorbidity and Excess Mortality Due to Natural Causes in Persons with Schizophrenia or Bipolar Affective Disorder. *PLoS One*. 2011;6(9):7. doi:10.1371/journal.pone.0024597
11. Laursen TM, Wahlbeck K, Hällgren J, et al. Life Expectancy and Death by Diseases of the Circulatory System in Patients with Bipolar Disorder or Schizophrenia in the Nordic Countries. *PLoS One*. 2013;8(6). doi:10.1371/journal.pone.0067133
12. Laursen TM, Wahlbeck K, Hällgren J, et al. Life expectancy and death by diseases of the circulatory system in patients with bipolar disorder or schizophrenia in the Nordic countries. *PLoS One*. 2013;8(6):e67133-e67133. doi:10.1371/journal.pone.0067133
13. Lomholt LH, Andersen D v, Sejrsgaard-Jacobsen C, et al. Mortality rate trends in patients diagnosed with schizophrenia or bipolar disorder: a nationwide study with 20 years of follow-up. *Int J Bipolar Disord*. 2019;7(1). doi:10.1186/s40345-018-0140-x
14. Medici CR, Videbech P, Gustafsson LN, Munk-Jørgensen P. Mortality and secular trend in the incidence of bipolar disorder. *J Affect Disord*. 2015;183((Medici C.R., Clara.reece.medici@post.au.dk; Gustafsson L.N.; Munk-Jørgensen P.) Aarhus University Hospital, Skovagervej 2, Risskov, Denmark(Videbech P.) Psychiatric Center Glostrup, Glostrup, Denmark):1-6. doi:10.1016/j.jad.2015.04.032
15. Mohamed MO, Rashid M, Farooq S, et al. Acute Myocardial Infarction in Severe Mental Illness: Prevalence, Clinical Outcomes, and Process of Care in US Hospitalizations. *Can J Cardiol*. 2019;35(7):821-830. doi:10.1016/j.cjca.2019.04.021
16. Newman SC, Bland RC. Suicide risk varies by subtype of affective disorder. *Acta Psychiatr Scand*. 1991;83(6):420-426.

<http://www.embase.com/search/results?subaction=viewrecord&from=export&id=L21203568>

1. Norton B, Whalley LJ. Mortality of a lithium-treated population. *The British Journal of Psychiatry*. 1984;145:277-282. doi:<http://dx.doi.org/10.1192/bjp.145.3.277>
2. Osborn DPJ, Levy G, Nazareth I, Petersen I, Islam A, King MB. Relative risk of cardiovascular and cancer mortality in people with severe mental illness from the United Kingdom’s general practice research database. *Arch Gen Psychiatry*. 2007;64(2):242-249. doi:<http://dx.doi.org/10.1001/archpsyc.64.2.242>
3. Osborn D, Levy G, Nazareth I, King M. Suicide and severe mental illnesses. Cohort study within the UK general practice research database. *Schizophr Res*. 2008;99(1-3):134-138. doi:10.1016/j.schres.2007.11.025
4. Ösby U, Brandt L, Correia N, Ekbom A, Sparén P. Excess mortality in bipolar and unipolar disorder in Sweden. *Arch Gen Psychiatry*. 2001;58(9):844-850. doi:10.1001/archpsyc.58.9.844
5. Y.-J. P. Widening gap in the excess mortality in elderly with bipolar affective disorder. *Int Psychogeriatr*. 2015;27((Pan Y.-J.) Department of Psychiatry, Far Eastern Memorial Hospital, Taiwan):S102. doi:10.1017/S1041610215002161
6. Y.-J. P, L.-L. Y, H.-Y. C, C.-K. C. Transformation of excess mortality in people with schizophrenia and bipolar disorder in Taiwan. *Psychol Med*. 2017;47(14):2483-2493. doi:10.1017/S0033291717001040
7. Park S, Rim SJ, Jo M, Lee MG, Kim CE. Comorbidity of Alcohol Use and Other Psychiatric Disorders and Suicide Mortality: Data from the South Korean National Health Insurance Cohort, 2002 to 2013. *Alcohol Clin Exp Res*. 2019;43(5):842-849. doi:10.1111/acer.13989
8. Ramsey CM, Spiraa AP, Mojtabai R, Eaton WW, Roth K, Leeb HB. Lifetime manic spectrum episodes and all-cause mortality: 26-year follow-up of the NIMH epidemiologic catchment area study. *J Affect Disord*. 2013;151(1):337-342. doi:10.1016/j.jad.2013.06.019
9. Saku M, Tokudome S, Ikeda M, et al. Mortality in psychiatric patients, with a specific focus on cancer mortality associated with schizophrenia. *Int J Epidemiol*. 1995;24(2):366-372. [http://ezproxy.libproxy.db.erau.edu/login?url=https://www.proquest.com/docview/77449624?acco](http://ezproxy.libproxy.db.erau.edu/login?url=https%3A//www.proquest.com/docview/77449624%3Facco) untid=27203
10. Schaffer A, Sinyor M, Reis C, Goldstein BI, Levitt AJ. Suicide in bipolar disorder: Characteristics and subgroups. *Bipolar Disord*. 2014;16(7):732-740. doi:10.1111/bdi.12219
11. Schneider B, Müller MJ, Philipp M. Mortality in affective disorders. *J Affect Disord*. 2001;65(3):263-

274. doi:10.1016/S0165-0327(00)00290-1

1. Schulman-Marcus J, Goyal P, Swaminathan R v, et al. Comparison of Trends in Incidence, Revascularization, and In-Hospital Mortality in ST-Elevation Myocardial Infarction in Patients with Versus Without Severe Mental Illness. *American Journal of Cardiology*. 2016;117(9):1405-1410. doi:10.1016/j.amjcard.2016.02.006
2. Sharma R, Markar HR. Mortality in affective disorder. *J Affect Disord*. 1994;31(2):91-96. doi:<http://dx.doi.org/10.1016/0165-0327(94)90112-0>
3. Tsuang MT, Woolson RF, Fleming JA. Causes of Death in Schizophrenia and Manic-Depression.

*British Journal of Psychiatry*. 1980;136(3):239-242. doi:DOI: 10.1192/bjp.136.3.239

1. Vinogradova Y, Coupland C, Hippisley-Cox J, Whyte S, Penny C. Effects of severe mental illness on survival of people with diabetes. *Br J Psychiatry*. 2010;197(4):272-277. doi:10.1192/bjp.bp.109.074674
2. Webb RT, Lichtenstein P, Larsson H, Geddes JR, Fazel S. Suicide, Hospital-Presenting Suicide Attempts, and Criminality in Bipolar Disorder: Examination of Risk for Multiple Adverse Outcomes. *J Clin Psychiatry*. 2014;75(8):E809-+. doi:10.4088/JCP.13m08899
3. Weeke A, Juel K, Væth M. Cardiovascular death and manic-depressive psychosis. *J Affect Disord*. 1987;13(3):287-292. doi:https://doi.org/10.1016/0165-0327(87)90049-8
4. Westman J, Hallgren J, Wahlbeck K, Erlinge D, Alfredsson L, Osby U. Cardiovascular mortality in bipolar disorder: a population-based cohort study in Sweden. *BMJ Open*. 2013;3(4):8. doi:10.1136/bmjopen-2012-002373
5. H.-H. Y, Westphal J, Hu Y, et al. Diagnosed mental health conditions and risk of suicide mortality.

*Psychiatric Services*. 2019;70(9):750-757. doi:10.1176/appi.ps.201800346
